# Structure and properties of tubular structures based on the quaternary misfit layered compound Sm_1−*x*_Y_*x*_S–TaS_2_†

**DOI:** 10.1039/d5ra00780a

**Published:** 2025-03-28

**Authors:** Simon Hettler, Mohammad Furqan, M. B. Sreedhara, Azat Khadiev, Reshef Tenne, Raul Arenal

**Affiliations:** a Instituto de Nanociencia y Materiales de Aragón (INMA), CSIC-Universidad de Zaragoza Zaragoza Spain hettler@unizar.es arenal@unizar.es; b Laboratorio de Microscopías Avanzadas (LMA), Universidad de Zaragoza Zaragoza Spain; c Solid State and Structural Chemistry Unit, Indian Institute of Science Bengaluru 560012 India; d Deutsches Elektronen-Synchrotron DESY Notkestr. 85 22607 Hamburg Germany; e Department of Molecular Chemistry and Materials Science, Weizmann Institute of Science Rehovot 7610001 Israel; f ARAID Foundation Zaragoza Spain

## Abstract

Misfit layered compounds (MLCs) are incommensurate super-lattices made up of two repeating subunits and are promising for different applications due to the possibility of a synergetic combination of the properties of the employed sublayers. A means of fine-tuning a specific MLC is to alloy one of the elements in the system. In this study, quaternary MLCs made of Sm_1−*x*_Y_*x*_S–TaS_2_ were successfully synthesized and the resulting structures were characterized in detail, with a specific focus on tubular structures. Owing to the small difference between the ionic radii of Sm and Y, the structure of the quaternary MLCs follows the one of pristine SmS–TaS_2_, irrespective of the degree of alloying and with an anisotropic change of the lattice parameters up to a maximum of 2.7%. Nevertheless, microscopy, spectroscopy, and diffraction techniques allow us to reveal the impact of the composition on important characteristics of the MLC. Notably, for a specific composition, the ratio between the lattice parameters of the two subsystems adopts a value of 3 to 5, which could lead to a transition toward a commensurate superstructure.

## Abstract 2

Nanotubes made of the quaternary misfit layered compound Sm_1−*x*_Y_*x*_S–TaS_2_ have been synthesized using the chemical vapour technique. Imaging, diffraction and spectroscopy shed light on their structure and properties.

## Graphical abstract

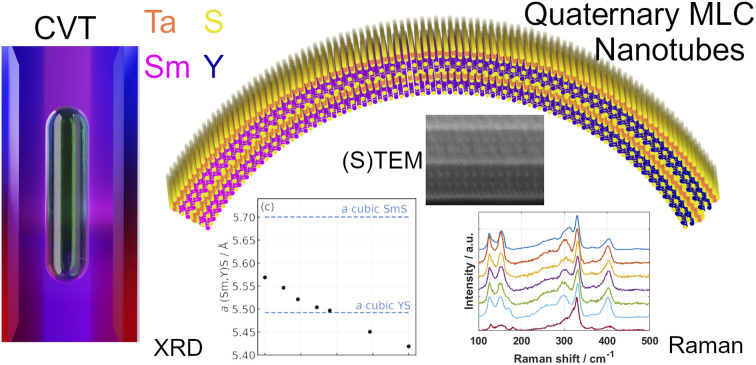

## 1 Introduction

**Fig. 1:**
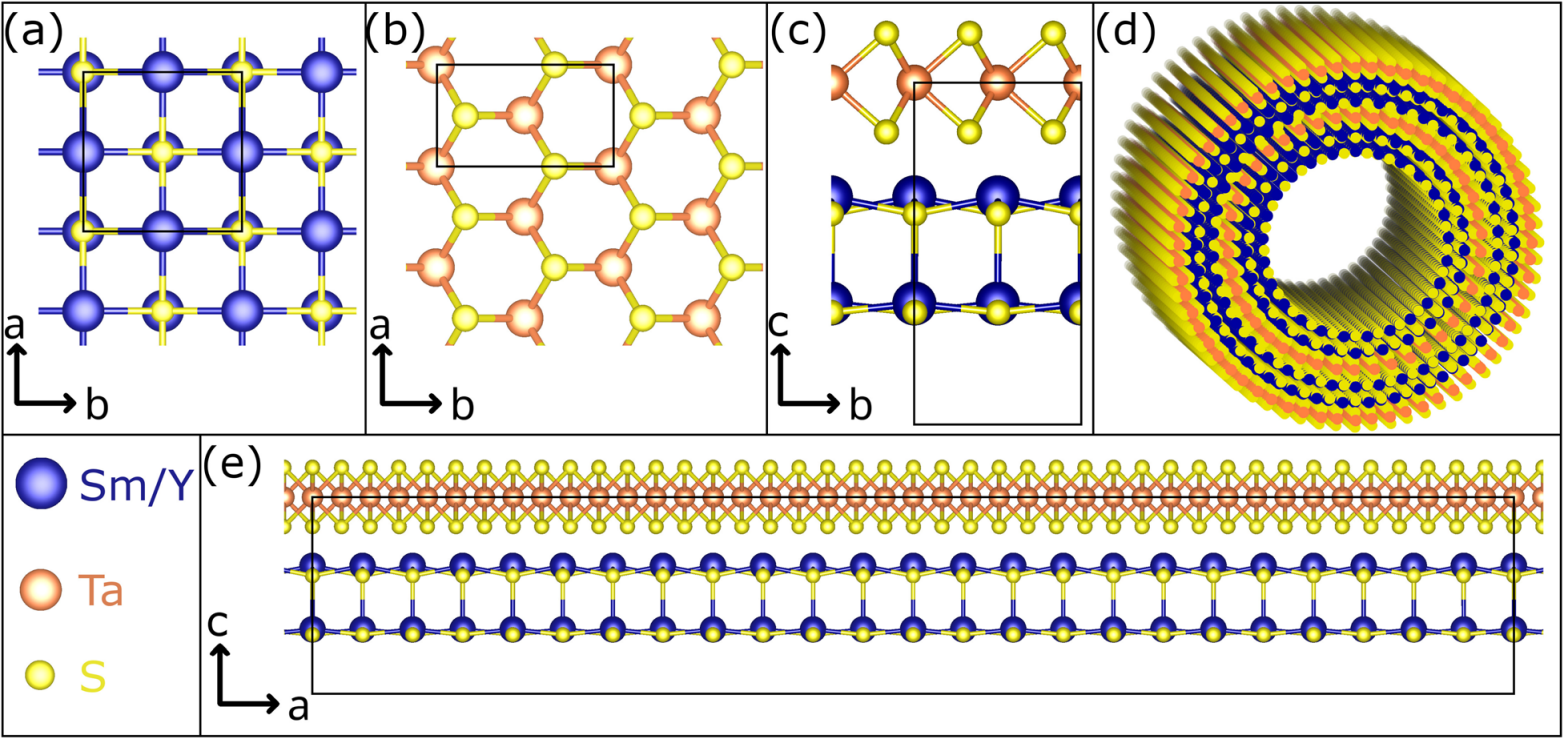
Schematic representation of the crystal structure of the Sm_1−*x*_Y_*x*_S–TaS_2_ MLC. (a) The Sm_1−*x*_Y_*x*_S subsystem crystallizes in a rock-salt lattice. (b) The hexagonal structure of TaS_2_ is seen in this view along the *c* axis. An orthorhombic unit has been indicated. (c) The view in the *c–b* plane reveals that both systems, stacked alternatively along the *c* axis, have similar lattice parameters in the *b* direction. (d) Sketch of a double-walled NT. (e) Due to the misfit in the *a* direction, an approximate super cell in the *c*–*a* plane adopts a large size. Here, 12 unit cells of (Sm,Y)S and 21 unit cells of TaS_2_ are depicted. It is noted that the marked unit cells in (c) and (e) correspond to half of the full unit cell in *c* direction of the MLC, which comprises two stacks.

Misfit layered compounds (MLCs) have garnered significant interest due to their distinctive, incommensurate crystallography and the possibility to combine two chemically diverse layered materials in a single compound.^1–6^ Within the realm of MLCs, those based on chalcogenides are particularly captivating, owing to their noteworthy electronic properties. While initial investigations focused on bulk materials, their tendency to bend due to inherent structural asymmetry led to the studies on micro- and nanosized tubules,^7,8^ mainly synthesized *via* the chemical vapor technique (CVT).^7,9–11^ Chalcogenide-based MLCs are composed of two layered chalcogenides arranged alternately along their *c*-axis. This stacking comprises a metal chalcogenide (MX) with a distorted rocksalt structure and a transition-metal dichalcogenide (TX_2_) with a distorted hexagonal structure (see Fig. 1 for a schematic presentation).^6,12–14^ Thereby, M represents elements such as Sn, Pb, Sb, Bi, or rare earth atoms (Ln), T represents metals like Sn, Ti, V, Cr, Nb, Ta and the chalcogen elements (S, Se, Te) are denoted by X. Due to the difference of the lattice parameters of the two subsystems in at least one direction, MLCs usually have an incommensurate structure. This is reflected by the chemical formula (MX)_1+*y*_(TX_2_)_*m*_ with the integer number *m* being the intercalation stage.^15,16^ An alternative designation for the stacking sequence of the MLC is O–T (stage 1) or O–T–T (stage 2), with O referring to the (distorted) rocksalt (MX) and T to the trigonal prismatic unit (TX_2_). The incommensurability between the *a*-axis of the MX and TX_2_ slabs is expressed as: 1 + *y* = 2*a*_T_/*a*_O_, with *y* taking values of 0.08 < *y* < 0.32. Recent density functional theory (DFT) calculations confirm the experimentally determined *y* value for (SnS)_1.17_NbS_2_.^17^ For simplicity, both *m* and *y* parameters are omitted throughout this manuscript, and the MLC structures are denoted as MX–TX_2_ or O–T. MLCs can also be regarded as intercalation compounds, whereby the MX (O) unit transfers charge to the hexagonal TX_2_ slab.

Micro- and nanotubes (NTs) made of MLCs hold a great promise for various applications, particularly in the field of thermoelectricity,^2,18–20^ which is attributed to the synergistic and complementary properties exhibited by the two layered compounds within the MLC structure. Being made of large atoms with many electrons, as well as unpaired spins, they tend to display complex many body phenomena at low temperatures, like superconductivity and Mott transition, highlighting their potential for quantum technologies. Furthermore, one could envision one sublattice (O) being ferromagnetic while the other slab is superconducting at low temperatures.^21^

A way to fine tune the properties of MLCs in a controllable way is to add a fourth element to the system and alloying either the M, T or X position in the MLC lattice. Alloying offers also the possibility to control the amount of charge transfer from the O to the T slab. Early on, *ab initio* calculations of lanthanum by strontium substitution in the lattice of LaS–CrS_2_ found a reduction of the amount of charge transfer between the two slabs of the MLC.^22^ More recently, *ab initio* calculations of lanthanum alloying in (PbSe)_1.14_(NbSe_2_)_2_ was reported and the lanthanum incorporation was found to shift the Fermi level of the MLC upwards, reducing its p-type conductivity.^23^ Another benefit of alloying the MLC nanotubes is ameliorating their yield.^9,24^ CVT has proven to be an efficient means to synthesize such quaternary MLCs in the form of NTs. Starting from LaS–TaS_2_,^25^ it has been shown that La can be readily alloyed by Sr or Y,^9,26^ while the substitution of Ta by Nb has proven to be more challenging.^27^ The high chemical selectivity of Se, binding exclusively to Ta, has led to asymmetric LaS–TaSe_2_ MLC NTs when partially replacing S by Se atoms.^28^

Replacing lanthanum by yttrium in LaS–TaS_2_ nanotubes (and flakes) induces significant changes in the MLC structural parameters and stoichiometry.^9^ The smaller Y^3+^ (ionic radius of 90 pm) compared to the La^3+^ (103 pm) leads to a smaller *a*-axis of the rocksalt unit cell (0.549 nm for YS compared to 0.581 nm for LaS). The interlayer distance *c*/2 is 1.09 nm for YS–TaS_2_ compared to 1.15 nm for the LaS–TaS_2_. The stoichiometry ratio (1 + *y*) varies from 1.14 in the lanthanum MLC compared to 1.20 in the pure yttrium compound. These changes were shown to influence the charge transfer and modulate the carrier density as vindicated by the redshift of the infrared (IR) plasmon cutoff upon replacing La^3+^ by Y^3+^.^9^ Related to LaS–TaS_2_, the MLC SmS–TaS_2_ in nanotubular form has been studied intensively in a recent investigation, revealing its superconducting phase below 5 K and giving insights into the interplay between the structure and electronic properties.^29^

In the present study, CVT has been used to alloy samarium (Sm) atoms by yttrium (Y) atoms yielding quaternary Sm_1−*x*_Y_*x*_S–TaS_2_ MLC structures for all values between *x* = 0 and *x* = 1. The resulting structures were studied by various microscopy, spectroscopy and diffraction techniques revealing a smooth replacement of Sm^3+^ by Y^3+^ and its impact on the MLC lattice. The results indicate a possible transition from the incommensurate MLC to a commensurate superstructure for a specific composition for which the incommensurability reaches a value that can be realized by a ratio of two integer numbers. Spectroscopic analyses reveal the possibility to fine tune the charge transfer and thus the electronic structure of the tantalum atoms in this quaternary MLC system.

## 2 Materials and methods

### 2.1 Synthesis of Sm_1−*x*_Y_*x*_S–TaS_2_ nanostructures

Sm_1−*x*_Y_*x*_S–TaS_2_ NTs (and flakes) were prepared by the CVT technique using evacuated quartz ampules following a well-established protocol used in previous studies.^9,28,29^ The reactants were mixed in a stoichiometric amount under inert atmosphere provided by a glovebox in order to prevent the oxidation of the precursors. Sm (Strem Chemicals 99.9%), Y (Strem Chemicals 99.9%), Ta (Alfa Aesar 99.9%), and S (Sigma-Aldrich 99.98%) were mixed in an agate mortar accordingly to the proportions 1 : 1 : 3 (19 mg (0.13 mmol) of Sm + Y; 25 mg (0.13 mmol) of Ta; and 13.2 mg (0.41 mmol) of S). 2–3 mg of TaCl_5_ (Sigma-Aldrich 99.99%) was added as a transport agent for the synthesis of nanotubular species. The ampules were sealed under vacuum (below 1 × 10^−5^ torr) provided by a rotary and a diffusion pump protected by a liquid N_2_ trap before being transferred to a pre-heated vertical furnace. In the following, a two-step annealing process with opposite temperature gradients, controlled by constant monitoring, between the top and the bottom of the furnace was applied. In the first step, a thermal gradient of 350 °C (bottom) and 800 °C (top) was applied during 1 h. The second step consisted of a 4 h annealing with an opposite gradient of 825 °C (bottom) and 400 °C (top). After the high-temperature annealing, the ampules were taken out of the furnace and naturally cooled down to room temperate. The product accumulated in the bottom part (hot zone) of the furnace was stored in a N_2_ atmosphere. For transmission electron microscopy (TEM) and Raman analyses, the powder was dispersed in ethanol by ultrasonication.

### 2.2 Analysis techniques

TEM and scanning (S)TEM analyses were conducted using two aberration-corrected Titan microscopes (Thermo Fisher Scientific). High-resolution (HR)TEM imaging and selected-area electron diffraction (SAED) were performed in an image-corrected Titan,^3^ while a probe-corrected Titan Low-Base, equipped with high-brightness gun (X-FEG), was employed for STEM imaging, STEM energy-dispersive X-ray spectroscopy (EDS) (Ultim X-MaxN 100TLE detector, Oxford Instruments) and monochromated low-loss electron energy-loss spectroscopy (EELS). All studies were conducted at an electron energy of 300 keV. Convergence angle was 25 mrad and acceptance angle for high-angle annular dark-field (HAADF)-STEM imaging 48 mrad and for EELS studies 2.3 mrad. STEM-EDS quantification was performed with the Aztec software (Oxford Instruments) using the Ta–M, Y–L and Y–M, Sm–L and S–K lines.

Scanning electron microscopy (SEM) was performed in an Inspect F50 and a Helios 650 (Thermo Fisher Scientific). The latter was also used to perform complimentary SEM-EDS and for focused ion beam (FIB) based TEM lamella preparation.

Raman spectroscopy was performed with a confocal Raman Alpha 300 M+ (WiTec) using a 633 nm laser operated at 0.5 mW power and a 50× objective. The spectrometer was operated with both the 600 and the 1800 grooves per mm grating. Samples were prepared by drop-casting the powder dispersions onto a glass substrate. Spectra were acquired for three individual NTs for each sample within a single session, except for pure YS–TaS_2_, for which previously published data was reused.^9^

The synchrotron powder X-ray diffraction (SPXRD) measurements were done at “P23 *in situ* X-ray diffraction and imaging beamline” at DESY PETRA III. The Sm_1−*x*_Y_*x*_S–TaS_2_ powder samples were placed inside the capillary under an inert Ar atmosphere inside a glove box and measured in transmission at 20 keV X-ray energy with an X-Spectrum Lambda GaAs 750 K detector while rotating the sample during the measurement. Azimuthal integration, as well as detector calibration, was done with the PyFAI software.^30^ Supplementary lab-based X-ray diffraction (XRD) was acquired from the as-synthesized powder in a PANalytical diffractometer Empyrean system using CuKα radiation.

## 3 Results and discussion

### 3.1 Morphology and chemical composition

**Fig. 2:**
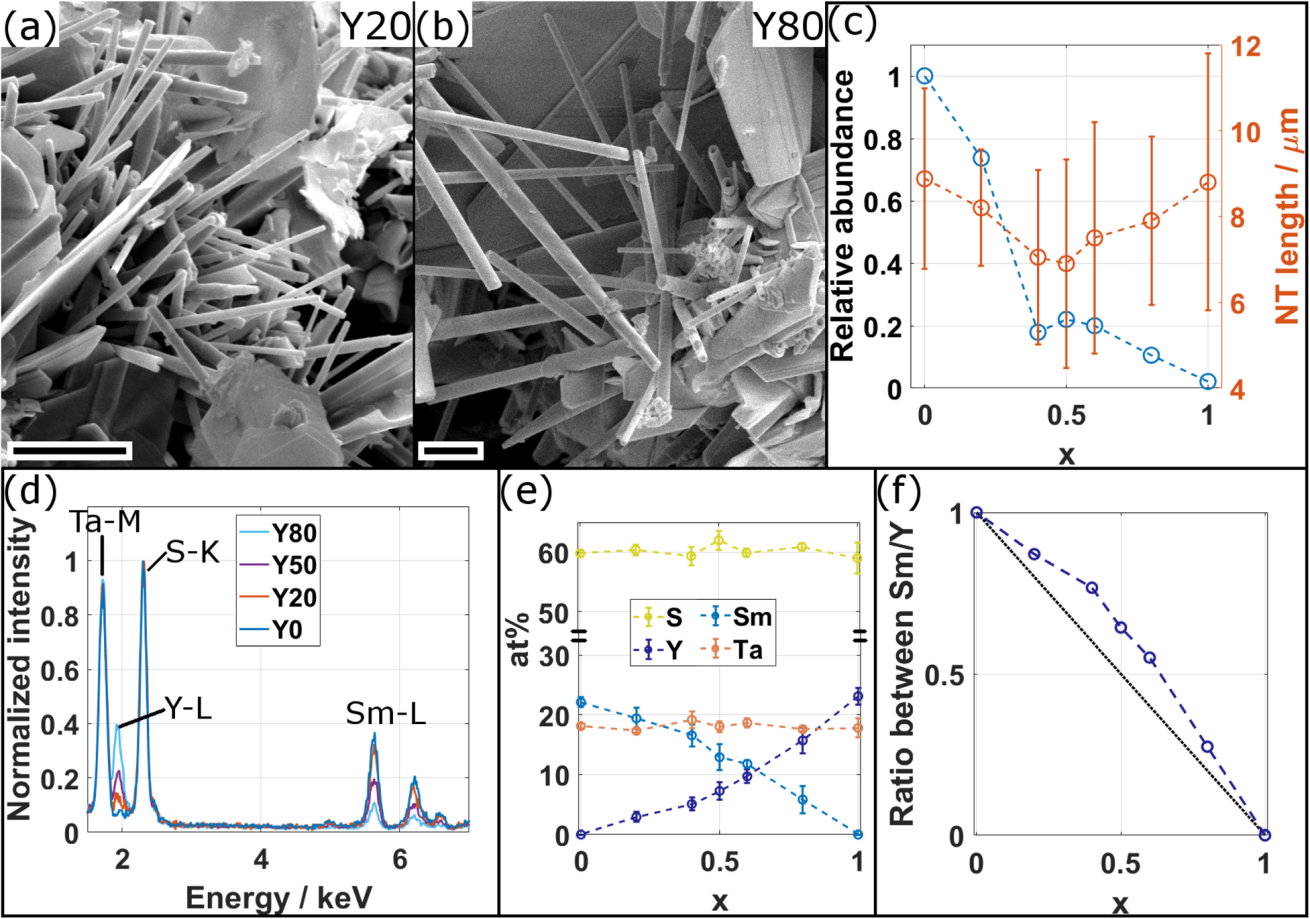
(a and b) SEM micrographs of the synthesis product for (a) the Y20 (*x* = 0.2) and (b) the Y80 (*x* = 0.8) specimens showing tubular structures together with scrolls and flakes. Scale bars are 2 μm. (c) Plot of the relative abundance with respect to *x* = 0 (blue circles) and the length of the tubular structures (orange circles). Dashed lines are guide to the eye. (d) Comparison of representative STEM-EDS spectra acquired from individual NTs for four different compositions as indicated. The increase (decrease) of intensity in the Y–L (Sm–L) edges with increasing *x* is evident, while intensity in Ta–M with respect to S–K (used for normalization) remains constant. (e) Average atomic composition of tubular structures in dependence of *x* confirms the qualitative observations made from (c) of a decrease/increase of Sm/Y with *x*. (f) Plot of ratio between Sm and Y in dependence of *x* (blue circles). Dashed blue line serves as guide to the eye and black dotted line represents expected ratio from the precursor composition.

Sm_1−*x*_Y_*x*_S–TaS_2_ MLCs were synthesized using the CVT technique for various yttrium fractions (*x* = 0, 0.2, 0.4, 0.5, 0.6, 0.8, 1) over the whole composition range and the corresponding samples are designated in the following as Y0, Y20, Y40, Y50, Y60, Y80, and Y100, respectively. SEM was used to analyze the morphology and abundance of the synthesis product. The SEM images reveal the presence of tubular and scrolled structures for all *x* with varying amount as exemplary depicted in Fig. 2a for the Y20 and Y80 sample. Similar SEM images for the other specimens are shown in the ESI, Section S1.† In addition to tubes and scrolls, MLC flakes are abundant and also non-MLC secondary phases were found, which are discussed below. The NTs exhibit varying lengths and wall thicknesses and can display a constant diameter along the tube or can possess a telescopic contour with varying diameters along the tube axis. Fig. S2 in the ESI† shows an assortment of TEM and STEM images providing examples for different morphologies of the synthesized structures and revealing the strong tendency to roll/bend of the MLC structures.

The size of fifteen tubes was measured for each composition and the average length and diameter were found to vary considerably with lengths between 1 and 14 μm (average of 8 μm) and diameters between 90 and 640 nm (average of 310 nm). A statistically significant variation with *x* of the length, diameter and the aspect ratio (length/diameter) could not be found, although the length seems to be the highest for pure specimens (*x* = 0, 1), as seen from Fig. 2c. The relative abundance of tubes with respect to *x* = 0 was determined by counting the number of tubes in the SEM images of the product. The highest abundance of tubular structures is found for pure SmS–TaS_2_ (*x* = 0) and is decreasing with *x* until reaching the minimum at *x* = 1 (Fig. 2c). In contrast to previous studies, in which a small amount of alloying led to an increase of the NT yield,^9,24^ the minimum used alloying degree (20%) in this study is relatively high and an increase of the yield could not be observed. The synthesis was conducted under conditions optimized for the synthesis of pure SmS–TaS_2_ NTs (825 °C at the hot side during the second annealing step)^31^ and the relative abundance will differ for varying conditions, which could be optimized for Y-containing MLCs.

The chemical composition of individual NTs was analyzed by STEM-EDS. Therefore, EDS data was averaged over a considerable portion of NTs that were identified to exhibit the MLC structure (see exemplary images in Section S3, ESI†). Fig. 2d shows a comparison of four exemplary spectra for different values of *x*, normalized to the intensity in the S–K peak. The comparison reveals a clear increase of the intensity in the Y–L line with *x*, while in parallel the intensity in the Sm–L line decreases and the intensity in the Ta–M remains largely unchanged with respect to the S–K used for normalization. The spectra for the entire energy range are shown in the ESI, Section S4,† which reveals an additional contribution from Cu and C, stemming from the TEM specimen support, and a negligible amount of oxygen. Fig. 2e displays the average elemental composition between the main constituents, revealing an approximately linear increase and decrease of the Y and Sm content with *x*, respectively. Ta and S contents remain largely constant as expected from the analysis of Fig. 2d. A look at the error bars, which only reflect the statistical error upon calculating the average composition of various tubes, indicates an increase in the spread of the Sm and Y content for mixed compositions with respect to Ta and S. This suggests that, within the same specimen, the Sm to Y composition slightly varies between individual NTs. Although the transition between Sm and Y seems to be almost linear with *x*, the plot of the ratio between Sm and Y (Fig. 2e) indicates a reduced incorporation of Y in the tubular structures as would have been expected from the precursor composition *x*. In the following, this ratio determined from EDS analysis, which better reflects the actual average composition of the synthesized NTs, denoted as *x*′, will be used as reference when plotting results obtained for the different specimens.

### 3.2 Crystal structure analysis

**Fig. 3:**
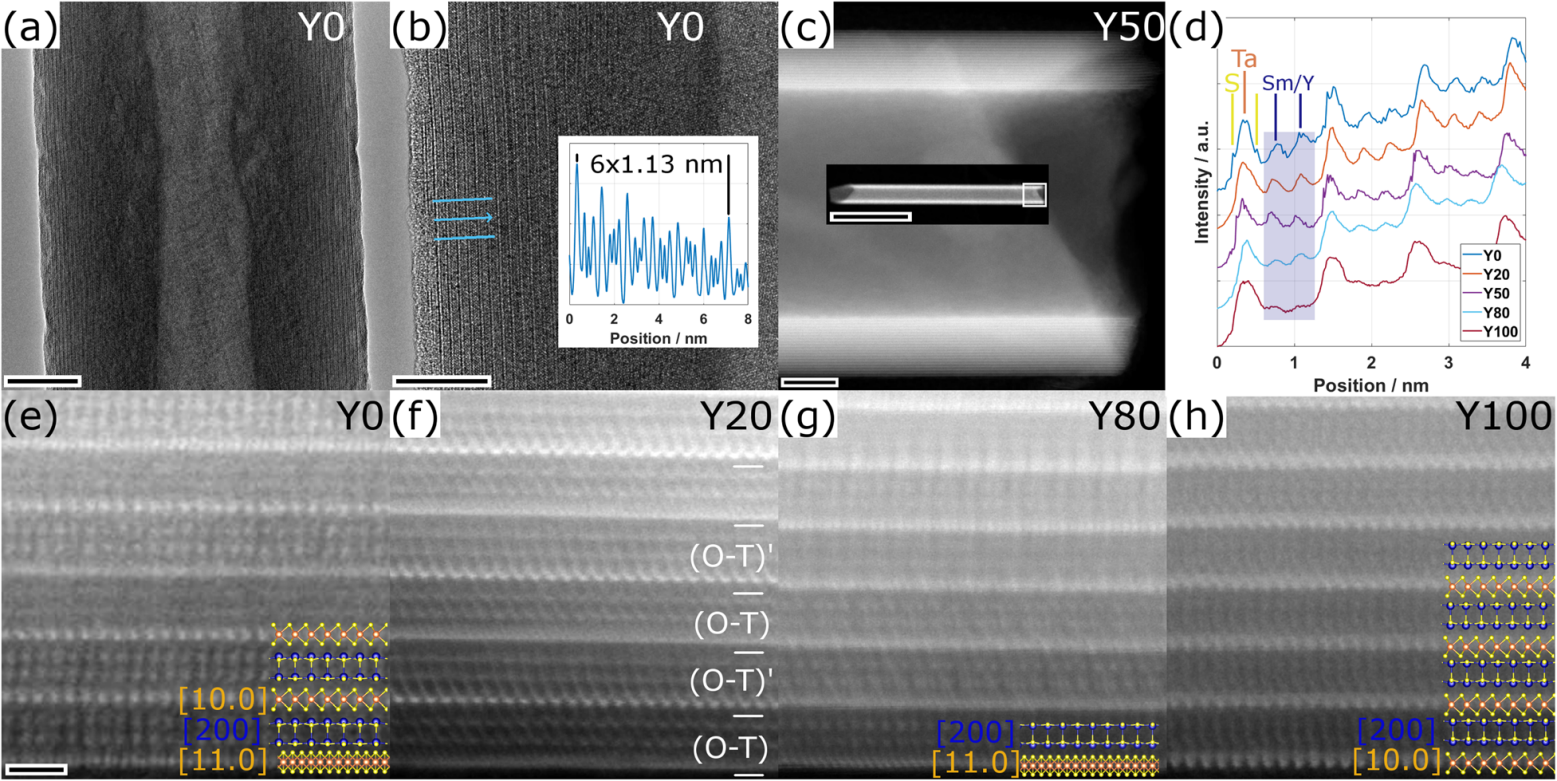
TEM and STEM analysis of the NTs. (a and b) TEM and HRTEM images of a NT of the Y0 specimen. (c) HAADF-STEM images of a Y50 specimen. White frame in the inset indicates where the main image was acquired. (d) Line profiles taken from the 4 outermost stacks from the HAADF STEM images shown in (e–h) and an additional one of the Y50 specimen. The Ta and Sm/Y atomic columns can be clearly distinguished for all the specimens. In some line profiles, the S atomic columns corresponding to the TaS_2_ structure are also resolved. The profiles were normalized with respect to the first Ta column and a vertical offset has been added for better visualization. (e–h) HAADF-STEM images of the 6 outermost layers of NTs for different specimens as indicated. Crystallographic orientations are indicated, orange and blue miller indices correspond to the TaS_2_ and (Sm,Y)S subsystems, respectively. Scale bars are (a) 20 nm, (b) 10 nm, (c) 20 nm (inset 500 nm), (e–h) 1 nm.

TEM and STEM imaging was applied to locally study the crystal structure of the synthesized structures at the atomic scale. Fig. 3a displays a TEM image of a NT of the Y0 specimen with outer and inner diameters of 90 nm and 20 nm, respectively. A HRTEM image obtained from the edge of the NT (Fig. 3b) reveals lattice fringes parallel to the edge and the stacking periodicity (*c*/2) is 1.13 nm as indicated by the line profile. This value agrees with the published data for such nanotubes.^32^ A very thin amorphous outer layer can be observed, which can be attributed to some surface contamination, possibly from organic residues remaining from the dispersion of the NTs in ethanol. Fig. 3c shows a HRSTEM image of a NT from the Y50 specimen with a length of 1.5 μm (inset image), an outer diameter of 90 nm and a wall thickness of 19 stacks (22 nm) on both sides, confirming the tubular structure.

Fig. 3e–h show four high-angle annular dark field STEM (HAADF-STEM) images of the six outermost walls of NTs from four specimens for different values of *x*, as indicated. The two different subsystems of the MLC structure are clearly revealed with the sheets with bright atomic columns corresponding to the tantalum atoms of the TaS_2_ subsystem sandwiched between two layers of sulfur atoms. In between the TaS_2_ sheets, two lines of atoms of the Sm_1−*x*_Y_*x*_S subsystem are observed. In several sheets of both subsystems, the electron beam coincides with a higher zone-axis direction and the individual atomic columns are resolved. Two different distances between adjacent tantalum atomic columns can be identified, which correspond to the [10.0] and the [11.0] crystallographic directions of the TaS_2_ structure, as indicated in the corresponding sheets for the different images. In the Sm_1−*x*_Y_*x*_S subsystem, only the [200] orientation can be identified in some of the sheets. The respective orientations between adjacent sheets differ with the composition of the NTs. The Y100 specimen (Fig. 3h) exhibits a single orientation for all the sheets as shown previously.^9^ In the Y20 specimen, the typical superstructure (O–T)–(O–T)′ seen in NTs of similar MLCs, where the orientation of the sheets repeats every second stack, is observed (Fig. 3f). In contrast, no superstructure or preferable orientation can be seen in the Y0 and Y80 NTs (Fig. 3e and g). Possible reasons for the formation or absence of the superstructure will be discussed below. A schematic visualization of the presence of different orientations of the layers and their impact on the HAADF-STEM signal is shown in Section S5, ESI.†

Line profiles were taken from the three outermost walls of the images shown in Fig. 3e–h and are compared with a similar one from the Y50 specimen in Fig. 3d. The atomic columns with higher intensity correspond to tantalum. In some profiles, two additional peaks linked to the adjacent sulfur atoms are resolved. The stack comprises two additional peaks in between the tantalum columns linked to the Sm_1−*x*_Y_*x*_S subsystem. The relative decrease with *x* of the intensity in the peaks linked to the Sm_1−*x*_Y_*x*_S columns compared to tantalum is clearly visible. This is explained by the dependence of intensity in HAADF-STEM images on the atomic number *Z*, which increases roughly following *Z*^1.7^. As *Z*_Sm_ = 62 is considerably higher than *Z*_Y_ = 39, the decreasing intensity confirms the increasing presence of Y in the Sm_1−*x*_Y_*x*_S subsystem with *x* and thus the smooth replacement of Sm by Y. Unfortunately, neither HAADF-STEM nor EDS could distinguish between specific samarium and yttrium atoms in the rocksalt lattice. This indifference can be possibly attributed to the considerable size (diameter) of the tubes and the presence of multiple atoms in each of the columns.

**Fig. 4:**
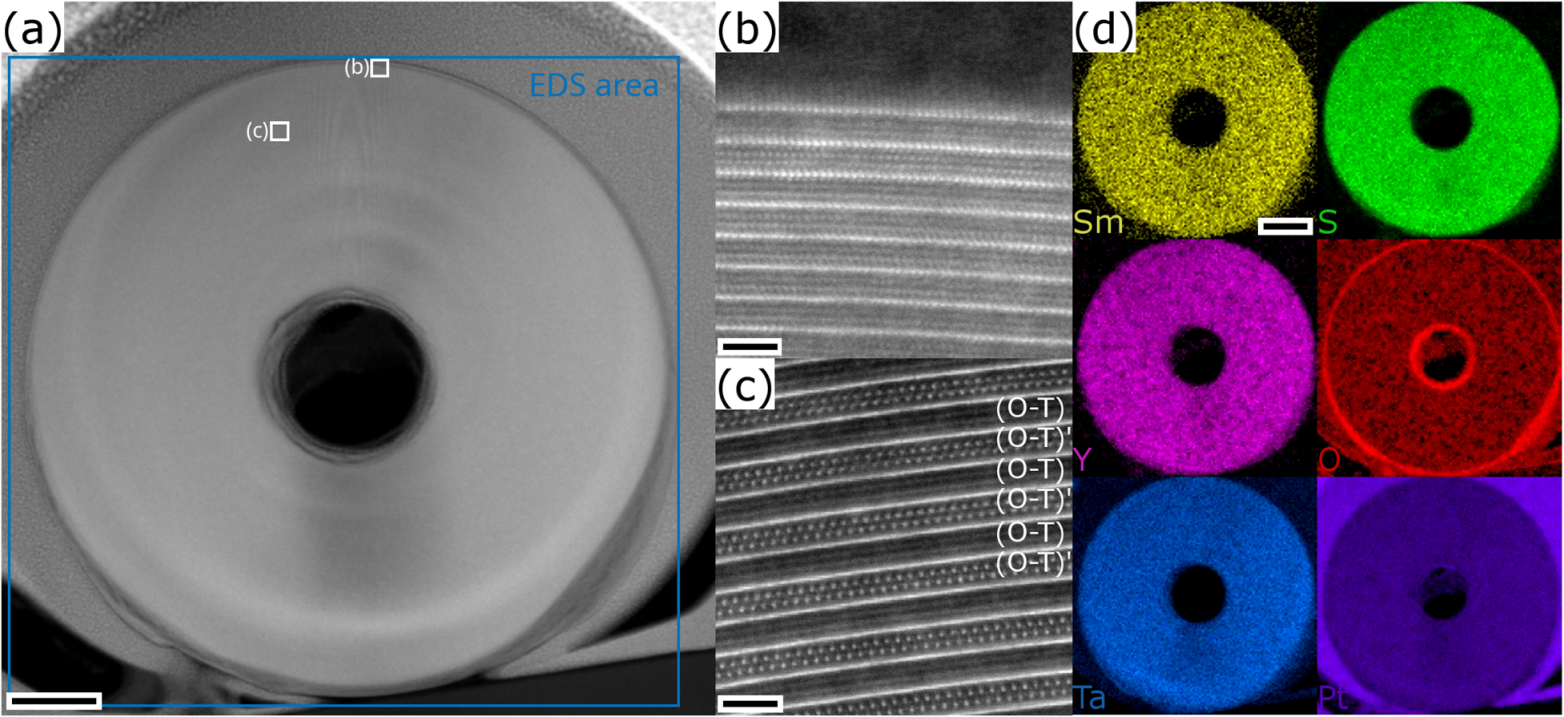
STEM imaging and EDS analysis of a cross-section FIB lamella of a Sm_0.2_Y_0.8_S–TaS_2_ NT. (a) Low magnification STEM-HAADF image with positions of STEM images (b and c) and area for EDS acquisition indicated. (b and c) STEM-HAADF images of different portions of the lamella showing varying orientations (b) and a two-stack superstructure in (c). (d) EDS elemental maps of the main constituents Sm, Y, Ta and S reveal a homogeneous distribution throughout the NT. Oxidation of inner and outer border is seen. Scale bars are (a) 70 nm, (b and c) 2 nm and (d) 100 nm, respectively.

To delve deeper into the structural and compositional details, thin cross-section lamellae of the NTs were prepared by the focused ion beam (FIB) technique. Fig. 4a illustrates a low-magnification STEM-HAADF image of a cross-sectional view of a tube prepared from the Y80 sample with an outer diameter of 530 nm. Different orientations and stacking sequences can be found throughout the NT as illustrated by the STEM-HAADF images in Fig. 4b and c. The typical (O–T)–(O–T)′ superstructure with alternating orientations is visible in Fig. 4c. EDS elemental maps were acquired to investigate the distribution of the main constituents throughout the cross section and the result is displayed in Fig. 4d. All four elements (Sm, Y, Ta and S) show a similar and homogeneous distribution. Similar analyses were performed for the Y50 and Y20 specimens, which revealed an identically homogeneous distribution. This indicates that the chemical composition of the MLC NT is independent of the radius of curvature of the walls.

**Fig. 5:**
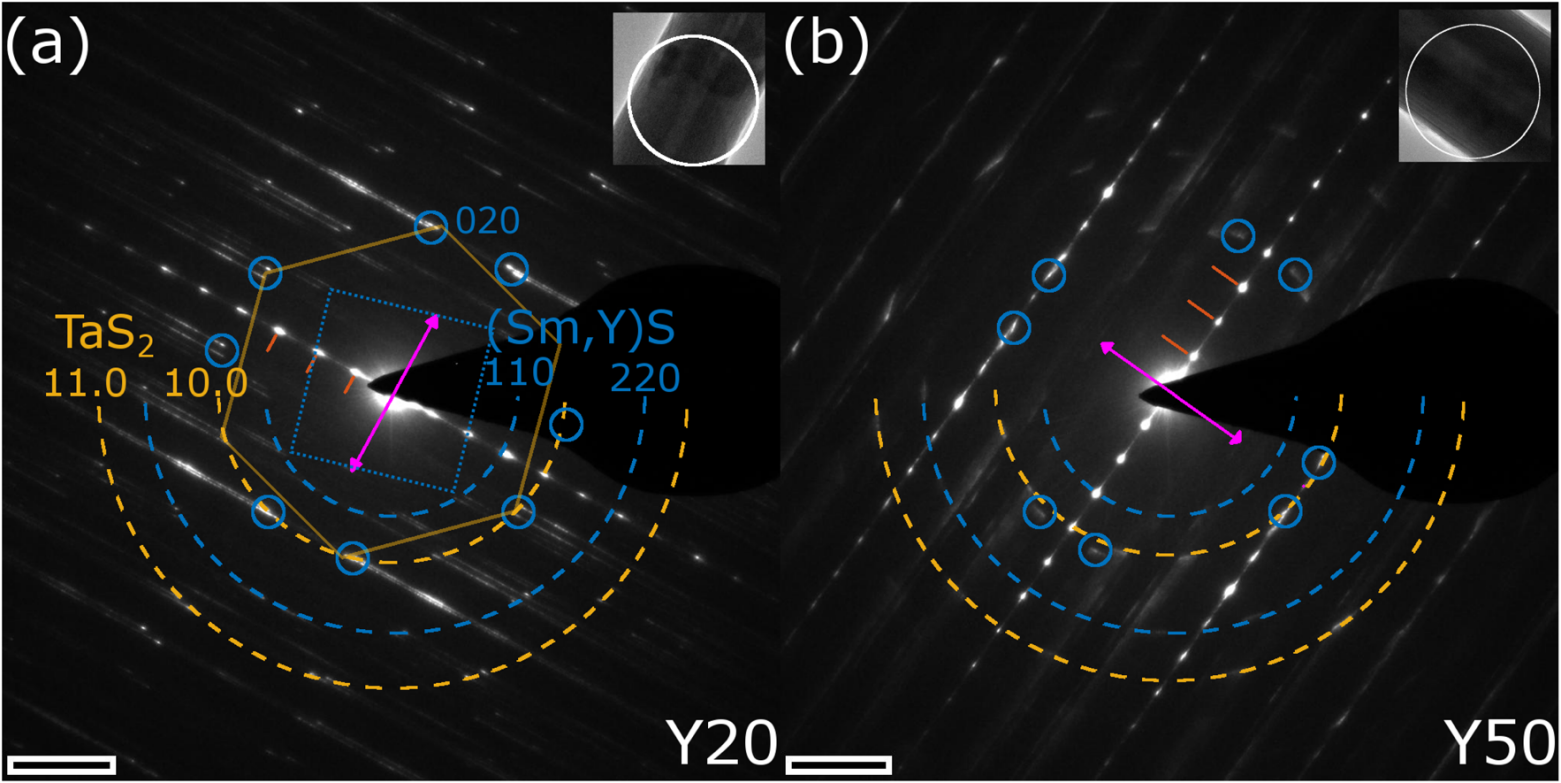
SAED analysis of the Sm_1−*x*_Y_*x*_S–TaS_2_ tubular structures. (a and b) SAED patterns obtained from an individual NT of the (a) Y20 and (b) Y50 samples. Insets show TEM images of the NTs with position of SA aperture indicated. The first three 00*l* reflections have been indicated by red lines, the TaS_2_ [10.0] and [11.0] reflections by dashed orange half circles, the (Sm,Y)S [110] and [220] reflections by blue half circles and the [200] and [020] reflections by small circles. The tube axis is seen from the inset image and reflected by the purple double arrow. Scale bars are 2 nm^−1^.

**Fig. 6:**
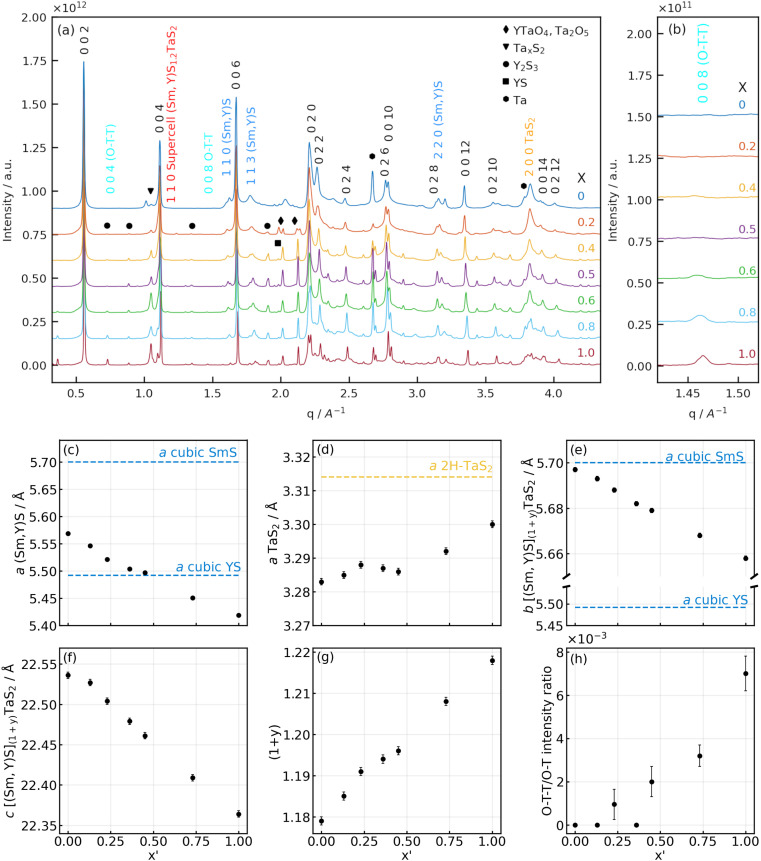
(a and b) X-ray diffractograms of Sm_1−*x*_Y_*x*_S–TaS_2_ powder samples synthesized with varying Y fraction (*x*) at different *q* ranges: (a) full-range diffractograms of the samples with (b) enlarged area of 008 (O–T–T) Sm_1−*x*_Y_*x*_S–TaS_2_ reflection. (c–h) Lattice parameters of [Sm_1−*x*_Y_*x*_S]_1+*y*_–TaS_2_ powder samples as a function of yttrium fraction found in the NTs (*x*′): *a* lattice parameters of the (c) (Sm,Y)S and (d) TaS_2_ subunits; common (e) *b* and (f) *c* parameters; (g) the incommensurability parameter (1 + *y*) between the *a*-axes of the (Sm,Y)S and TaS_2_ slabs and (h) the ratio of 008 (O–T–T) and 002 (O–T) reflections. Literature values for cubic SmS,^33^ YS^34^ and 2H-TaS_2_ ^35^ have been indicated as horizontal lines.

The chemical analysis together with the imaging at the local scale thus confirms the formation of NTs made of the quaternary MLC Sm_1−*x*_Y_*x*_S–TaS_2_. Further structural analysis was obtained by diffraction techniques, whose results are shown in Fig. 5 and 6. Selected-area electron diffraction (SAED) patterns were obtained for several individual NTs for the different specimens. Fig. 5a and b show exemplary patterns for the Y20 and Y50 specimens, respectively. The reflection spots visible in the spectra can be divided into three categories: Firstly, the stacking periodicity along the *c*-axis gives rise to strong [00*l*] spots with 1.1 nm lattice distance (marked by red lines) perpendicular to the tube axis (pink double arrow), which can be inferred from the inset TEM images. Secondly, the reflections of the first two orders ([10.0], 0.28 nm and [11.0], 0.16 nm) of the TaS_2_ subsystem are marked by orange dashed half circles. The reflections with six-fold symmetry can be attributed to its (distorted) hexagonal lattice. As an example, one such set of six reflections is marked by a hexagon in Fig. 5a. Both SAED patterns show two sets of six reflections indicating the presence of two distinct orientations, or folding vectors, of the TaS_2_ throughout the entire tube. Thirdly, reflections are observed for the Sm_1−*x*_Y_*x*_S subsystem, which are indicated by blue dashed half circles ([110], 0.39 nm and [220], 0.195 nm) and small blue circles indicate the [200] and [020] crystal directions. These reflections are 4-fold symmetric, due to the (distorted) cubic crystal structure of the Sm_1−*x*_Y_*x*_S subunit as indicated by a dotted square in Fig. 5a. Here too, two sets of four reflections are seen in both patterns, which have a defined angle with respect to the TaS_2_ lattice orientation. This angle is 30° in the pattern shown in Fig. 5a, which is most commonly observed in similar MLC NTs and leads to the (O–T)–(O–T)′ superstructure directly observed also by imaging in Fig. 3f and 4c. In this case, all eight [200] reflections coincide with TaS_2_ [10.0] reflections. The situation is different in Fig. 5b, where the relative angle is only approximately 20°, which is best observed from the marked [200] reflections of the (Sm,Y)S subsystem. In this case, only four of the [200] reflections coincide with TaS_2_ [10.0] reflections, the ones pointing parallel to the tube axis. The other four [200] reflections are not located on the dashed orange half circle, but at slightly larger spatial frequencies leading to a smaller lattice distance of 0.27 nm, being 4% smaller compared to the perpendicular direction (0.28 nm). This illustrates the importance of the relative angle between the orientations of the two subsystems. While the Sm_1−*x*_Y_*x*_S subsystem adopts a largely symmetric lattice in the *a*–*b* plane if the angle is 30° such as seen in Fig. 5a, additional misfit stress at different angles causes it to adopt an asymmetric, stretched lattice (Fig. 5b).

A systematic measurement of the corresponding distances of the *c*-axis, TaS_2_ and (Sm,Y)S reflections was conducted and the result is given in Section S6 of the ESI.† No statistically significant variation of the *c*-axis (1.1 nm) and TaS_2_ periodicities ([10.0] = 0.28 nm and [11.0] = 0.16 nm) could be found, while the Sm_1−*x*_Y_*x*_S [110] reflections show a minor decrease from 0.39 nm in the case of Y0 to 0.38 nm for the Y100 specimen. This minimum change of only 3% is explained by the very similar lattice constants of the SmS and YS crystal structures of 0.57 nm (high-pressure phase^33^) and 0.549 nm,^34^ respectively. Additional SAED patterns for the whole set of specimens are shown in the ESI, Section S7.† The case with two folding vectors shown in Fig. 5a and b is the most common one. However, in the studied specimens, a considerable number of different NTs exhibited multiple folding vectors. A single folding vector is frequently encountered in the Y100 specimen, as reported previously^9^ and also visible from the image in Fig. 3h, while rarely seen in other compositions.

Lab-based X-ray diffraction (XRD) and synchrotron-based powder (SP)XRD analysis were performed on the raw powder to obtain information on the crystal structure on a macroscopic scale. It should be borne in mind that the analyzed samples contain both NTs and flakes of (Sm,Y)S–TaS_2_. As the SPXRD data offers significantly higher precision, we focus here on those results. Fig. 6a compares the SPXRD patterns for the different specimens. The position of the main diffraction peaks can be ascribed to the orthorhombic lattice of SmS–TaS_2_ (*Fm*2*m* space group^3^) consisting of (Sm,Y)S and TaS_2_ sublattices having *b* and *c* axes parallel and equal in length with incommensurability occurring along the *a* axis direction.^3^

The diffractograms of all the specimens show strong reflections common for both sublattices: the set of 00*l* reflections, which correspond to the stacking periodicity of the layers, as well as 0*k*0 and 0*kl* reflections that are attributed to the common *b* axis. These reflections are marked by black indices in Fig. 6a. The *hk*0 and *h*00 diffraction peaks attributed to the in-plane periodicity of (Sm,Y)S (dark blue indices) and TaS_2_ (yellow indices) sublattices are less intense and have a high degree of asymmetry due to the curvature of the nanotubes, as seen in Fig. 6a. This set of reflections was used to determine the lattice parameters of the samples synthesized with varying yttrium fractions. Although the 0*kl* reflections are expected to be present only in the MLC flakes and absent in nanotubes,^31^ we assume that the lattice parameters of the MLC flakes will be similar to MLC nanotubes within the same sample.

The results of the lattice parameter refinement are presented in Fig. 6c–h and Section S8, ESI.† From Fig. 6c, e and f, it is seen that the *a* parameter of the (Sm,Y)S, as well as the *b* and *c* parameters of the common lattice, decreases with yttrium doping. The variation of the lattice parameters shows a linear behavior following Vegard's law^36^ and indicates the gradual substitution of samarium atoms by yttrium. The decrease of the lattice parameters with increasing yttrium fraction is attributed to the difference in ionic radii of Sm (96 pm) and Y (90 pm),^37^ a similar effect was already reported in alloyed MLC nanotubes^9,32^ and MLC crystals.^38–40^ It is instructive to compare the amount of lattice shrinking for the different parameters. While the lattice parameter of the common *b* axis is closer to the literature value of SmS and only shrinks by 0.7% between *x* = 0 and *x* = 1 (Fig. 6e), the *a* parameter of the (Sm,Y)S decreases by 2.7% and even below the literature value of YS. This observation indicates that the stress generated by the common *b* axis is released in the incommensurate *a* axis.

Since both (Sm,Y)S and TaS_2_ lattices in MLC are interconnected *via* the *b* axis, it is interesting to observe the changes of the *a* parameter of TaS_2_ (Fig. 6d). Due to yttrium alloying, the *b* parameter of the common axis compresses in the TaS_2_ subunit, and TaS_2_ has to release the strain by expanding the *a* lattice parameter. The change in the *a* parameter of TaS_2_ is only about 0.5% between SmS–TaS_2_ and YS–TaS_2_ specimens and such a minor expansion cannot be resolved by SAED measurements. Importantly though, the *a* parameter of TaS_2_ gradually grows with the Y fraction up to 0.23, then there is a plateau up to 0.53 before it starts to grow again (Fig. 6d). The calculation of the unit-cell area in the *a*–*b* plane of TaS_2_ in orthorhombic representation, given by *b*_MLC_ × *a*_TaS2_, is instructive to assess the overall strain in the TaS_2_ lattice. Interestingly, this area is constant (0.187(1) nm^2^) and does not vary statistically significant with *x*′ (Section S8, ESI†). This suggests that the generated stress due to the shrinking common *b* axis is completely released within the incommensurate *a* axis.

Since the samples show a relatively small *a*-TaS_2_ parameter change (approx. 0.5%) and considerable change (approx. 2.7%) in *a*-(Sm,Y)S lattice parameter due to the substitution of samarium atoms by yttrium, one can also calculate the incommensurability between the MX and TX_2_ slabs in [(Sm,Y)S]_1+*y*_–TaS_2_ as 
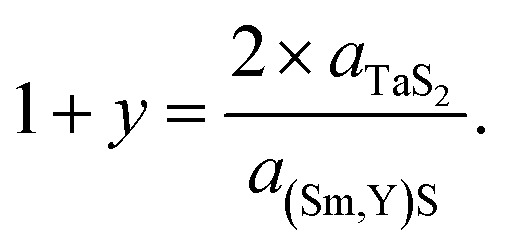
 The results of the calculations are shown in Fig. 6g and Section S8, ESI:† the (1 + *y*) of SmS_1+*y*_–TaS_2_ is 1.18 being close to the 1.19 observed in (SmS)_1.19_–TaS_2_ MLC crystals,^3^ then it gradually grows up to 1.218 in YS–TaS_2_, which is close to a previously reported value.^9^

As was shown above, it is possible to control the incommensurability between (Sm,Y)S and TaS_2_ sublattices by Y substitution, and one could imagine the situation when the total structure could become commensurate. Indeed, in some samples, the value of (1 + *y*) is very close to 1.2, which can be realized by an integer number of unit cells, *i.e.*, 5 unit cells of TaS_2_ and 3 unit cells of (Sm,Y)S. In such a combination, a supercell structure with *a* = 5 × *a*(TaS_2_), *b* = *b*([(Sm,Y)]_(1+*y*)_TaS_2_), *c* = *c*([(Sm,Y)]_(1+*y*)_TaS_2_) parameters could appear that would signify a transition from an incommensurate to a commensurate structure. Two results of the XRD data analysis suggest that such a supercell could indeed be present within the samples with specific Sm/Y ratios. Firstly, the plateau observed in the *a* parameter of TaS_2_ between *x*′ = 0.25 and 0.5 (Fig. 6d), for which *a*(TaS_2_ = 3.29 Å) and (1 + *y*) is closest to 1.2, could be caused by the presence of a commensurate phase. Secondly, a diffraction peak that would correspond to the 110 plane of the supercell with an interplanar distance of 5.365(6) Å is visible for the mentioned range of values of *x* (shown in Fig. 6a). A detailed view of the peak and a description of the concept of such a possible supercell structure are presented in Section S9, ESI.†

Interestingly, a commensurate structure with a similar 3 : 5 unit cell relation was also found in the La_1.2_CrS_3.2_ ^39,41,42^ misfit compound, where such commensurability was seen from supercell reflections with the help of SAED. Although commensurate phases were already observed in a few MLCs,^39,41–44^ further experimental data, as well as a possible application of the superspace approach,^45^ are required to corroborate the existence of a commensurate phase and analyze its structural properties, which is out of the scope of this manuscript.

At higher (1 + *y*) values, less TaS_2_ is required to form the MLC; thus, an excess of TaS_2_ is expected to form in the reaction mixture. Additionally, reflections linked to binary YS (black square in Fig. 6a) and to D-Y_2_S_3_ ^46^ (black circles) are observed, which increase in intensity with *x* and thus increase the excess of TaS_2_. Probably, this is the reason for the (O–T–T) structure formation at high Y fractions (Fig. 6b and h), where the MLC stack comprises two slabs of TaS_2_ per one (Sm,Y)S slab in the compound having the [(Sm,Y)S]_1+*y*_–[TaS_2_]_2_ formula. As the 004 (O–T–T) reflection overlaps with a reflection of D-Y_2_S_3_, the 008 (O–T–T) is used to track the evolution of (O–T–T) with *x* and *x*′ in Fig. 6b and h. The *c* parameter of the (O–T–T) phase also becomes smaller with Y alloying (Section S8, ESI†), thus showing that in both (O–T) and (O–T–T) yttrium atoms substitute samarium. It is also seen that the diffraction peak around 1.05 Å^−1^ (black triangle in Fig. 6a), which is attributed to the tantalum sulfides, is growing with the Y content and supports the assumption mentioned above. Moreover, the appearance of Y-rich phases explains the sub-representation of Y in the alloyed MLC NTs as would have been expected from the precursor composition (Fig. 2f). In some of the specimens, sharp peaks are present at positions close to metallic Ta, which have been marked by black hexagons, suggesting that parts of the precursor material did not take part in the synthesis reaction.

The XRD analysis also reveals other secondary phases detected in the products. These phases give rise to several peaks, mainly in the range of *q* = 1.7–2.1 Å^−1^. Secondary phases have been detected by SEM imaging to adopt mostly rod-like morphologies (see Section S10, ESI†) and SEM-EDS analysis indicates that these structures are based on Y, Ta and S (little to no Sm) and are (partially) oxidized. A clear identification of known crystal structures based on these constituents could not be achieved and will require further, more detailed investigation; possible candidates are YTaO_4_ and Ta_2_O_5_ (black diamond symbols in Fig. 6a). The formation of these secondary phases could be minimized by finely adjusting the synthesis temperatures to optimize the yield of tubular MLC structures for a specific composition.

In general, the results obtained from the SPXRD analysis are in line with the aforementioned SAED measurements, *e.g.* the (Sm,Y)S interplanar spacings and the *c* axis periodicity (Section S8, ESI†). Considering the accuracy of both measurement techniques, the complexity of the system manifested by broad diffraction peaks and the fact that the SPXRD analysis comprises both flakes and NTs, the agreement between the two methods is more than satisfactory.

### 3.3 Electron energy-loss spectroscopy

**Fig. 7:**
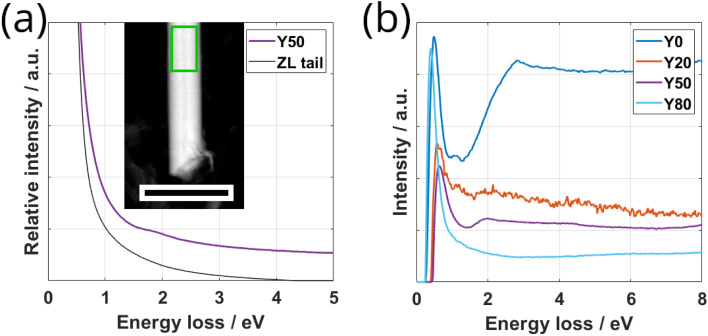
Low-loss EELS analysis of Sm_1−*x*_Y_*x*_S–TaS_2_ NTs. (a) Low-loss EELS spectrum obtained from the central part of a Y50 NT as marked by the green frame in the inset DF STEM image (scale bar 400 nm). The solid purple line represents the raw spectrum and the black line depicts the zero-loss tail mirrored from the energy gain side. (b) Comparison of spectra obtained from individual NTs for different values of *x*, after subtraction of the mirrored zero-loss tail. Y0 and Y80 spectra were slightly scaled by a factor of 1.2 and 0.8 to better visualize the changes in the spectra.

Monochromated electron energy-loss spectroscopy (EELS) in STEM mode permits investigating the low-loss response in the IR-regime. EELS data of SmS–TaS_2_ NTs was recently presented,^29^ showing a strong difference between the response of pure 2H-TaS_2_ and the MLC structure. A strong peak was found around 1 eV for TaS_2_, which was attributed to an intra-atomic transition within the 5d_*z*^2^_ band of Ta. This peak is absent in the MLC, which can be attributed to the filling of the band by charge-transfer from the SmS layer. In the present study, we acquired low-loss EELS data for various values of *x*. This analysis allows to investigate a possible modification of the low-loss response with the alloying degree.^9,28,47^ The solid purple line in Fig. 7a shows such a spectrum for a Y50 NT, normalized with respect to the sum intensity in the entire spectrum. In addition, the zero-loss tail from the energy-gain side (negative energy loss) has been mirrored around 0 eV and is plotted as black line in Fig. 7a. For a better comparison of the spectra acquired for different specimens, the spectra were background-subtracted with this mirrored zero-loss tail, similar as done.^29^

Fig. 7b shows the four background-subtracted spectra of individual NTs of the Y0, Y20, Y50 and Y80 specimens. The absence of the strong peak at 1 eV visible in 2H-TaS_2_ is observed in all spectra as expected from the general presence of charge transfer in MLCs. The spectrum for *x* = 0 (pure SmS–TaS_2_) corresponds well with the previous work,^29^ showing a minor peak at 1 eV followed by an increase in intensity. With increasing *x*, the minor peak at 1 eV first further diminishes and slightly shifts to higher energies for *x* = 0.2 before disappearing completely for *x* = 0.5 and above. The onset of increasing intensity is maintained up to *x* = 0.5 but also disappears for *x* = 0.8, which shows an almost flat curve. This comparison indicates that the electronic properties of the MLC electronic states in the IR region can be fine-tuned by alloying.

In addition to the IR region, the shape of the bulk plasmon of the tubular structures for different values for *x* in comparison to TaS_2_ was studied using TEM in diffraction mode. The results are presented in Section S11, ESI† and are summarized here: the bulk plasmon of the MLC structures exhibits a considerable broadening compared to the sharp peak observed in TaS_2_, which is attributed to the change in crystal structure leading to a higher damping of collective electron excitations, specifically in the *c* direction of the MLC. The plasmon energy *E*_P_ of TaS_2_ and for some values of *x* of the Sm_1−*x*_Y_*x*_S–TaS_2_ MLC was determined by fitting a Drude function following the free-electron model^48^ and the value is found to be similar to the one for TaS_2_ (22 eV). *E*_P_ is found to exhibit a minimum for mixed composition, which again indicates a change in the electronic band structure with the degree of alloying.

### 3.4 Raman spectroscopy

**Fig. 8:**
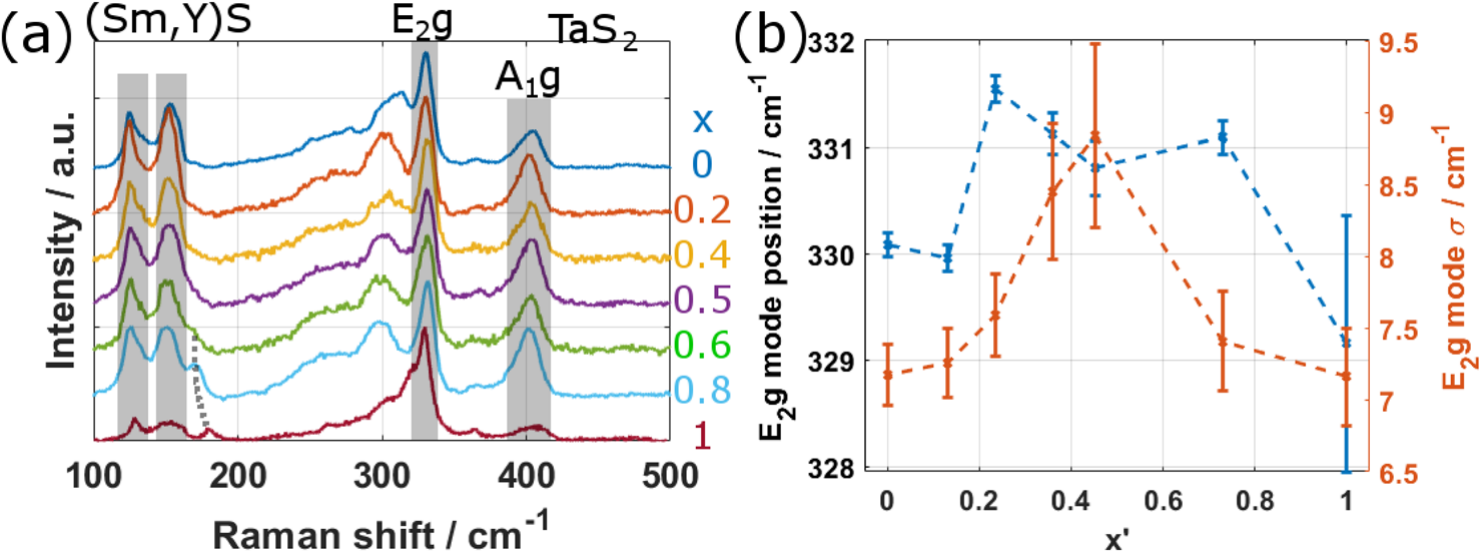
Raman analysis of the specimens. (a) Comparison of Raman spectra obtained by averaging three spectra from different NTs for each value of *x*. The RSI and II modes of the (Sm,Y)S subsystem, an additional peak for small values of *x* (dotted gray line) and the E_2g_ and A_1g_ modes of TaS_2_ are resolved. The spectrum for *x* = 0 was acquired in a different instrument and with a different objective. (b) Plot of the E_2g_ mode position and E_2g_ mode width *σ* in dependence of *x*′.

Raman spectroscopy can give detailed information on both the electronic and vibrational states of crystalline materials. We systematically investigated the Raman response by acquiring spectra from different individual tubes for all values of *x* using a 633 nm laser. The spectra of the NTs belonging to specimens with *x* < 1 (*i.e.* all except YS–TaS_2_) have been acquired within a single session to allow for a detailed quantitative analysis. Fig. 8a shows a comparison of the Raman spectra, obtained by averaging the spectra taken from three different tubes of each specimen in comparison with the spectra of pure YS–TaS_2_ obtained in our previous study.^9^ The spectra have been normalized with respect to the peak at 330 cm^−1^ and are shown for a larger spectral range in Section S12, ESI,† indicating the absence of oxygen-based modes. All spectra exhibit similar features, independent of *x*, which generally agrees with the Raman analyses of different MLC compounds, including individual NTs, that have been previously reported.^7,9,49^

The Raman signal in the region between 100 and 500 cm^−1^ can be divided into two parts linked to the two subsystems. Two A_1g_ modes are seen for the Sm_1−*x*_Y_*x*_S subsystem at 127 and 152 cm^−1^, which are designated as rocksalt RSI and RSII modes. Despite the strong difference between the masses of Sm (150u) and Y (89), the position of these peaks does not vary significantly with *x*. However, both peaks slightly broaden with *x*, indicating that the Raman signal for mixed composition is dominated by the Sm atoms. In addition, a new peak arises for *x* = 1 at approximately 180 cm^−1^, which gradually shifts downwards in energy and decreases in intensity for decreasing *x*, before disappearing and turning into a shoulder for *x* < 0.6 (black dashed line in Fig. 8a). These observations agree with the results obtained from NTs of the related MLC (La,Y)S–TaS_2_. However, the peak at 180 cm^−1^ could not be identified in the previous work.^9^ Recent results on the Raman response of 2H-TaS_2_ in dependence of the thickness of the crystal revealed a second-order peak at 180 cm^−1^,^50^ which might be the origin of the peak seen in the studied NTs of MLCs with *x* ≥ 0.8.

The part located at higher Raman shifts is related to the second subsystem TaS_2_. Two peaks can be identified at approximately 330 and 400 cm^−1^, which correspond to the E^1^_2g_ and the A_1g_ modes of TaS_2_, respectively. The E^1^_2g_ mode is strongly shifted with respect to bulk 2H-TaS_2_ (280 cm^−1^). This shift has been attributed to the charge transfer from the LnS subunit.^49^ We analyzed the E^1^_2g_ peak position and width in detail by fitting a single Gaussian peak to the E^1^_2g_ mode. The result is displayed in Fig. 8b, where the peak position (blue crosses) and the peak width (orange crosses) are plotted over *x*′. A peak broadening for mixed compositions is clearly observed, which could be attributed to two effects. Firstly, the averaging of the Raman spectra from three different tubes could lead to the broadening as the actual composition between the studied NTs could vary slightly, leading to small shifts. Secondly, and more probably, it could be attributed to the introduced local inhomogeneity in the system due to alloying. Regarding the position of the E^1^_2g_ mode (blue crosses in Fig. 8b), a minimum is found for pure compositions (*x* = 0,1) and it shifts to higher energies for mixed compositions. If the mode position depended only on the charge transfer, one would expect a linear transition between *x* = 0 and *x* = 1, which is however not the case. The observed shift of the position suggests thus a more complex interplay between the Sm/Y composition and the inelastic Raman scattering, which depends on both the phonon dispersion and the electronic states, requiring further studies. Another observation that suggests a difference in Raman scattering between pure and alloyed systems is a comparison of the intensities of the E^1^_2g_ mode of TaS_2_ on one side and the RS modes as well as the A_1g_ mode of TaS_2_ on the other side. The relative intensity in the E^1^_2g_ mode is considerably higher for *x* = 1 (YS–TaS_2_) and also in reduced strength for *x* = 0 (SmS–TaS_2_) compared to mixed compositions. As the E^1^_2g_ mode corresponds to an in-plane or shear vibration mode, while A_1g_ (for both RS and TaS_2_) to an out-of-plane or radial breathing mode, the results suggest the preferred excitation of in-plane modes for pure MLCs, while alloying increases the relative intensity of out-of-plane modes. This suggests that the disorder at the atomic level in alloyed MLCs favors the interlayer interaction, while interlayer interaction seems to be reduced for pure MLCs. A recent study of the thickness dependence of the E^1^_2g_ mode in 2H-TaS_2_ revealed a trend of the Raman response when going from bulk to monolayer that is similar to our observation when going from bulk to MLC.^50^ The position of the E^1^_2g_ mode shifts to 300 cm^−1^ for monolayer 2H-TaS_2_, larger than the bulk value (280 cm^−1^), but less than for MLC (330 cm^−1^) and also, the intensity in the E^1^_2g_ increases with respect to the intensity in the A_1g_ modes. This suggests that the 2H-TaS_2_ in intercalated form present in MLCs resembles more closely its monolayer than bulk form.

## 4 Conclusion

In this study, tubular structures from the family of the quaternary misfit layered compound (MLC) Sm_1−*x*_Y_*x*_S–TaS_2_ were synthesized by the chemical vapor technique and analyzed using different microscopic, spectroscopic and diffraction techniques. The experimental results reveal that the samarium atoms in the SmS subsystem could be replaced by yttrium atoms for all studied values of *x*, although the amount of incorporated samarium was somewhat higher than expected from the precursor composition. Due to the very similar crystal structure of SmS and YS, the commensurate MLC lattice parameter *b* is found to shrink by only 0.7% for *x* = 1 compared to *x* = 0, while the incommensurate (Sm,Y)S lattice parameter *a*(Sm_1−*x*_Y_*x*_S) shows a linear decrease by 2.7%. The incommensurate TaS_2_ lattice parameter *a*(TaS_2_) shows an increase by 0.5%, which is attributed to strain relaxation and which leaves the TaS_2_ unit cell area in the *a*–*b* plane constant. The non-linear change of the MLC lattice parameters with *x* and a weak superstructure reflection possibly indicate an incommensurate–commensurate phase transition for the range of Y doping ratios *x* = 0.2–0.6.

As reported previously,^9^ the two subsystems in the MLC structure of pure YS–TaS_2_ nanotubes exhibit a single orientation with a commensurate *b*-axis coinciding with the tube axis. In the quaternary Sm_1−*x*_Y_*x*_S–TaS_2_ tubes (*x* > 0), the layers mostly adopt a superstructure, where adjacent stacks along the *c*-axis exhibit a fixed relative rotation by 30° with respect to each other. The composition was observed to be homogeneous within the cross section of individual NTs, irrespective of the diameter (and the radius of curvature) of the wall. Spectroscopic analysis by electron energy-loss spectroscopy and Raman scattering indicate a complex interplay between the alloying degree on side and the charge transfer and thus the electronic structure as well as the phonon configuration on the other side, giving the possibility to fine tune the MLC properties by alloying the metal in the rock-salt unit of the MLC. Spectroscopic and structural analyses suggest that the characteristics of the quaternary compound is dominated by the heavy samarium atoms even for higher contents of yttrium in the MLC.

## Data availability

The raw data supporting this article are available on Zenodo under the following link: https://doi.org/10.5281/zenodo.15070391.

## Author contributions

Investigation: SH, MF, MBS, AK – formal analysis: SH, AK – supervision: RT, RA – writing original draft: SH, MF, AK – writing review & editing: all.

## Conflicts of interest

There are no conflicts to declare.

## Supplementary Material

RA-015-D5RA00780A-s001
