# Supplementary material for: Structure and properties of tubular structures based on the quaternary misfit layered compound Sm1−xYxS–TaS2
Source: RSC Adv. 2025 Mar 28;15(13):9605–17. doi: 10.1039/d5ra00780a (PMC11952113; doi:10.1039/d5ra00780a)
Supplement: RA-015-D5RA00780A-s001 [file RA-015-D5RA00780A-s001.pdf]

Supplementary Information (SI) for RSC Advances.
This journal is © The Royal Society of Chemistry 2025

Supplementary Information for

Structure and Properties of Tubular Structures based on the Quaternary Misfit Layered Compound $Sm_{1-x}Y_xS-TaS_2$

Simon Hettler[1,2,^], Mohammad Furqan[1,2], MB Sreedhara[3], Azat Khadiev[4], Reshef Tenne[5] and Raul Arenal[1,2,6]

1. Instituto de Nanociencia y Materiales de Aragón (INMA), CSIC-Universidad de Zaragoza, Zaragoza, Spain
2. Laboratorio de Microscopias Avanzadas (LMA), Universidad de Zaragoza, Zaragoza, Spain
3. Solid State and Structural Chemistry Unit, Indian Institute of Science, Bengaluru, India
4. Deutsches Elektronen-Synchrotron DESY, Notkestr. 85, 22607 Hamburg, Germany
5. Department of Molecular Chemistry and Materials Science, Weizmann Institute of Science, Rehovot, Israel
6. ARAID Foundation, Zaragoza, Spain

^. Present address: Laboratorium für Elektronenmikroskopie, Karlsruher Institut für Technologie, Karlsruhe, Germany

Corresponding authors e-mails: hettler@unizar.es, arenal@unizar.es

Content

S1: SEM images
S2: STEM/TEM morphology
S3: STEM-EDS acquisition
S4: EDS spectra comparison entire range
S5: Schematic illustration of relative layer orientations
S6: Lattice spacings SAED
S7 Additional SAED patterns
S8: SPXRD lattice parameter refinement and comparison with SAED
S9: Incommensurate-commensurate transition
S10: SEM-EDS secondary phase
S11: Bulk plasmon analysis
S12: Raman spectra

S1. SEM images

Representative SEM images for the different synthesis products for all investigated specimens.

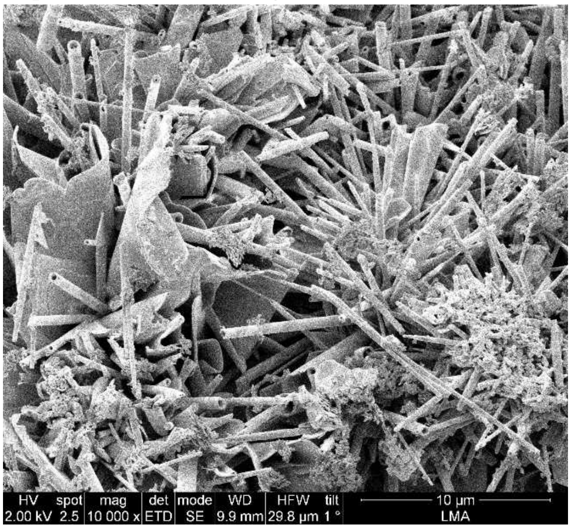

Y0

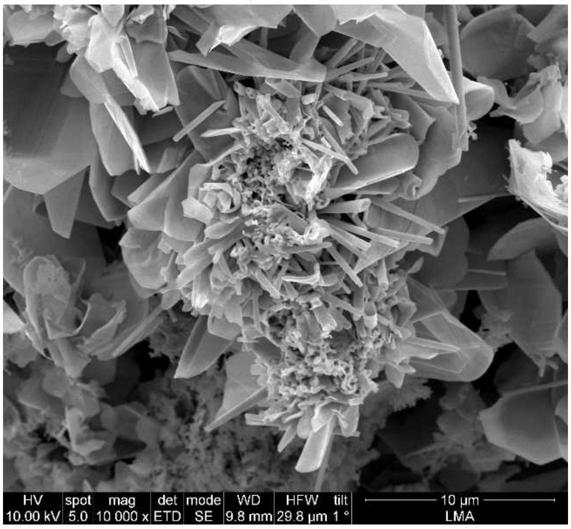

Y20

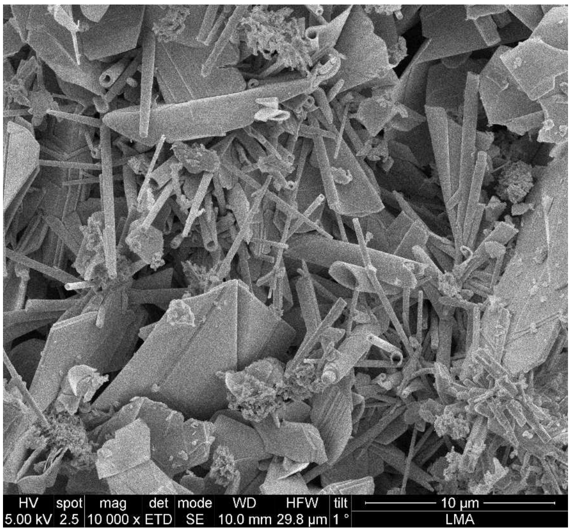

Y40

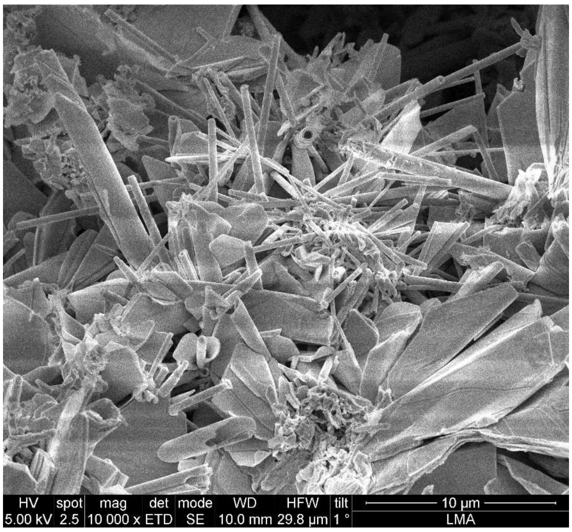

Y50

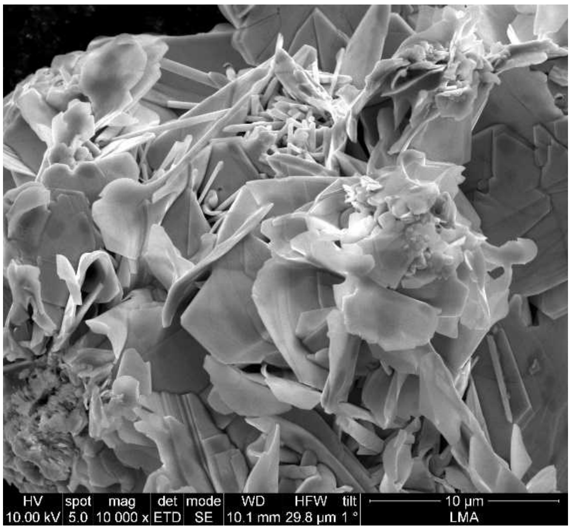

Y60

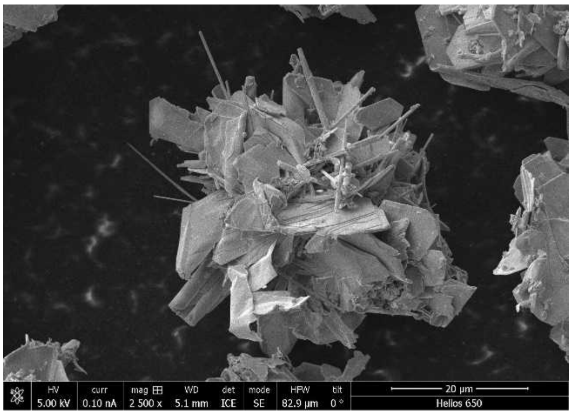

Y80

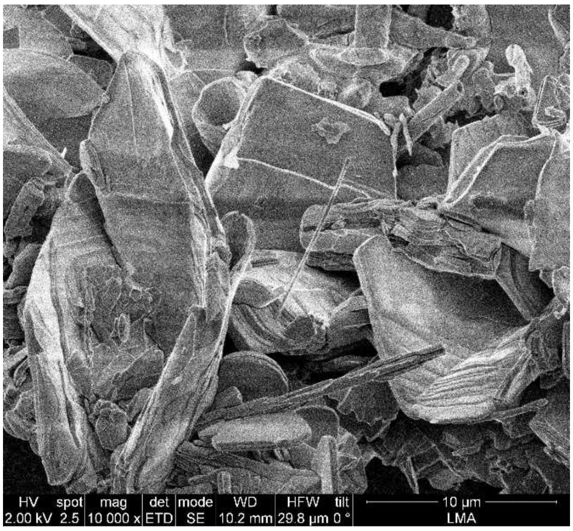

Y100

S2. STEM/TEM images morphology

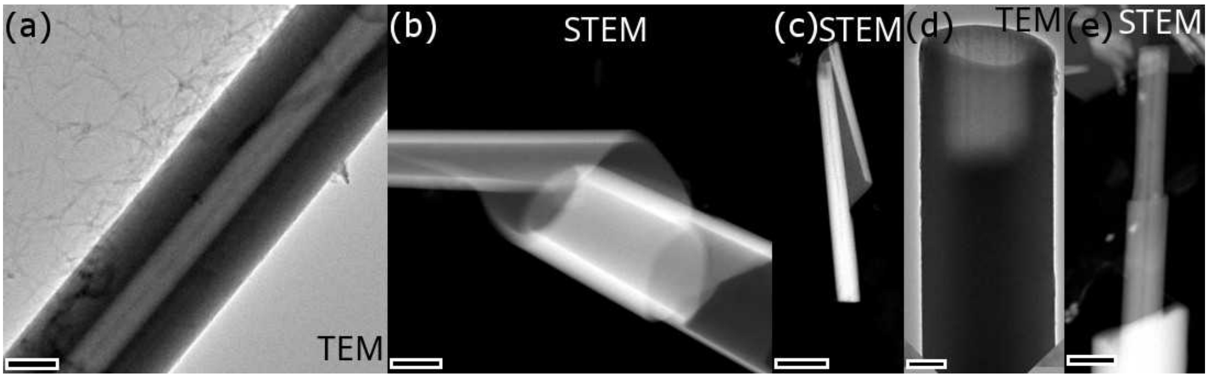

(a) Regular tubular structure. (b,c) Two tubes/scrolls connected by a sheet. (d) Tube with filled lower part and hollow end. (e) Telescopic tube. The imaging technique has been annotated in each image. Scale bars are (a,c) 60 nm, (b) 200 nm and (d,e) 70 nm.

S3. STEM-EDS acquisition

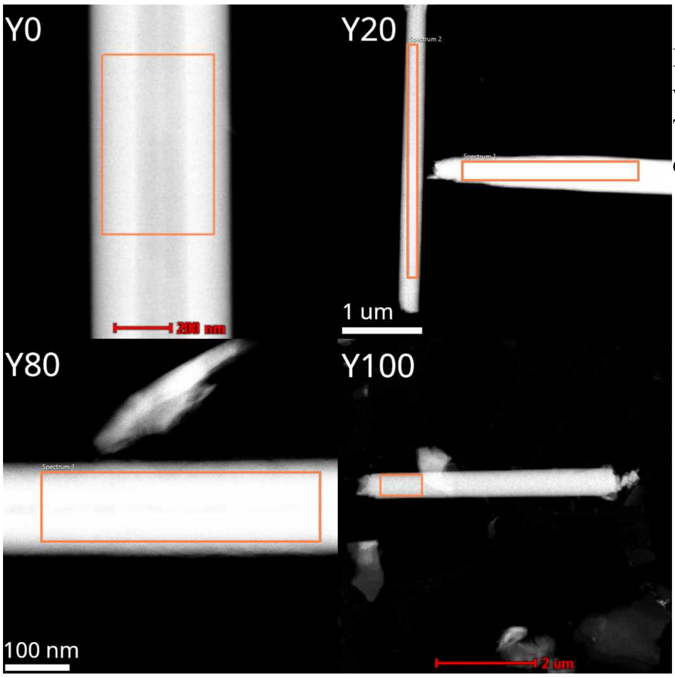

Four exemplary HAADF-STEM images with windows of STEM-EDS acquisition marked. The two windows shown for the Y20 NTs correspond to two individual measurements.

S4. EDS spectrum comparison entire range

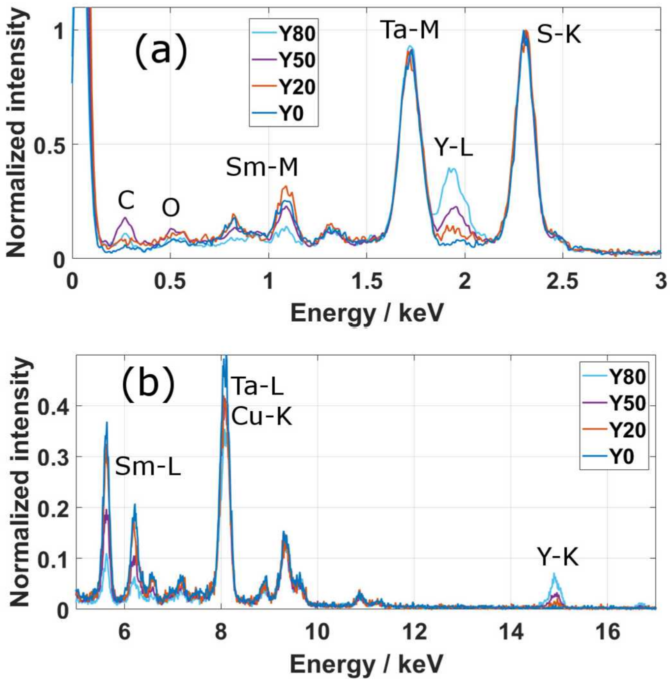

Comparison of the spectra shown in Figure 2d for the whole energy range with (a) covering the lower energy and (b) the higher energy portions, respectively.

S5. Schematic illustration of relative layer orientations

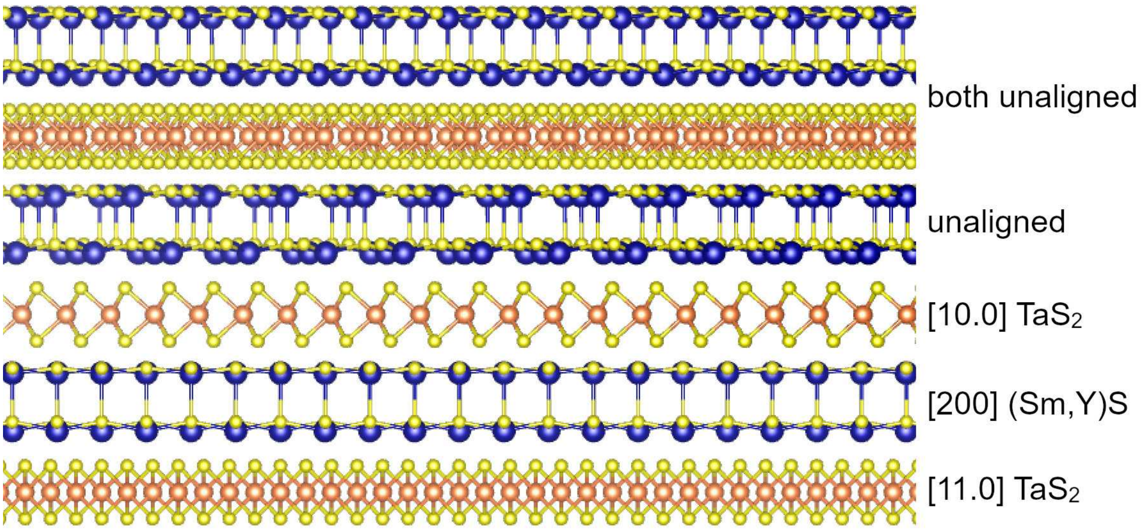

The varying appearance of the atomic contrast in HAADF-STEM images of the two different subsystems (Figure 3e-h) is explained by their relative orientation with respect to the propagation direction of the electron beam. The schematic exemplary shows three possible orientations, with the upper stack being unaligned, leading to no distinguishable contrast. In the second stack, only the TaS_2 layer exhibits an ordered alignment while in the lowest, both are aligned.

S6. Lattice spacings obtained from SAED

Lattice spacings obtained from SAED analysis

x	x`	(Sm,Y)S-TaS_2 c/2 (nm)	(Sm,Y)S 110 (nm)	TaS2 10.0 (nm)	(Sm,Y)S 220 (nm)	TaS2 11.0 (nm)	(Sm,Y)S 200 a-b asymmetry / %
0	0	1.105	0.388	0.279	0.197	0.162	1.47
0.2	0.13	1.112	0.388	0.281	0.197	0.162	0.21
0.5	0.36	1.101	0.385	0.281	0.195	0.162	2.07
0.8	0.73	1.112	0.381	0.280	0.196	0.162	1.28
1	1	1.105	0.381	0.280	0.191	0.162	

S7. Additional SAED patterns

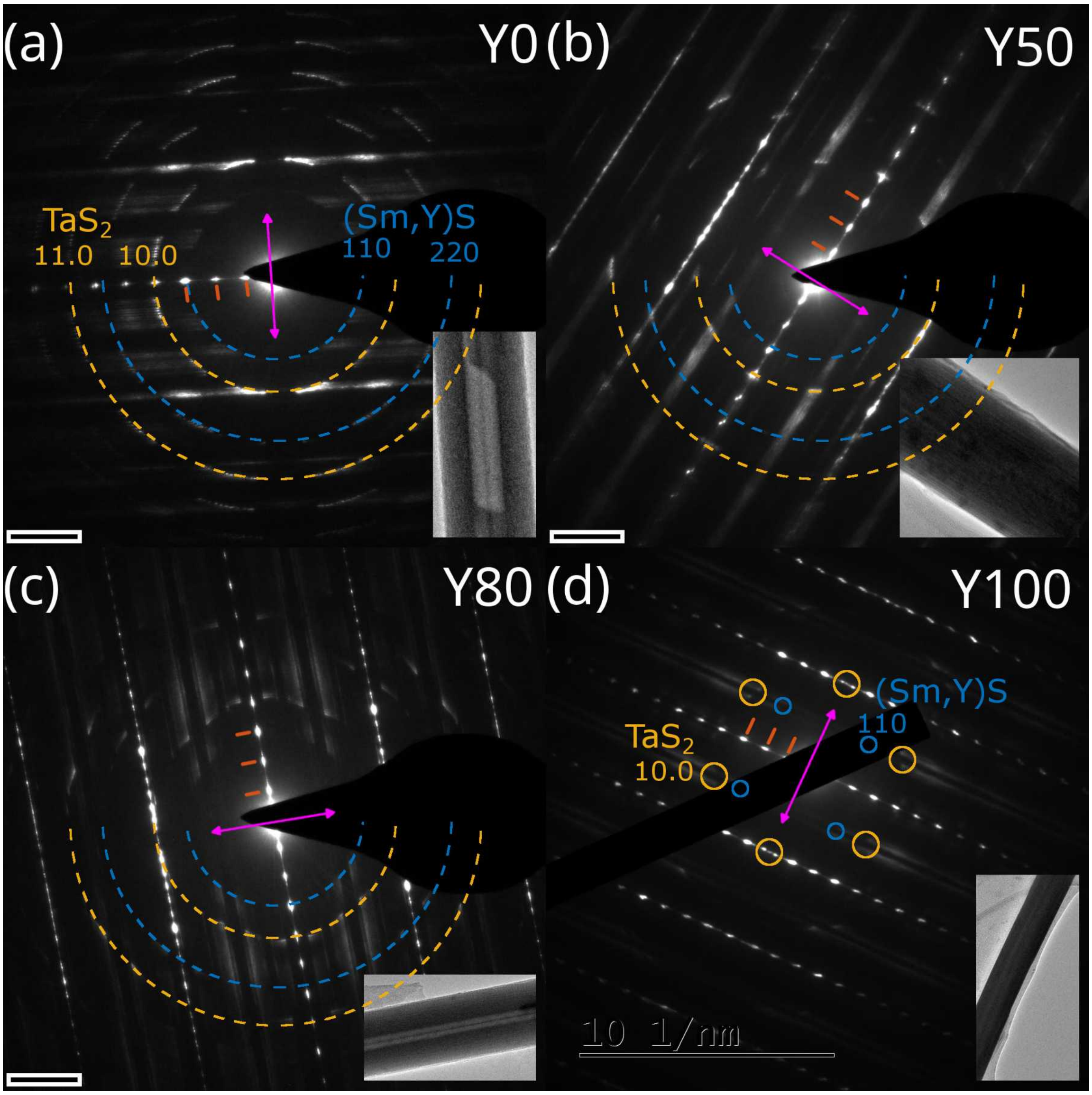

Exemplary SAED patterns and inset TEM images obtained from individual NTs from the (a) Y0, (b) Y50, (c) Y80 and (d) Y100 specimens. TaS_2 and (Sm,Y)S reflection rings have been marked in (a-c), where multiple folding vectors are visible in (a), while the single set of 4 and 6 reflections has been marked in the case with single folding vector seen in (d). C-axis reflections and tube axis have been marked. The SAED pattern in (b) is a special case, exhibiting one major folding vector and an additional one with strongly reduced intensity. Scale bars are (a-c) 2 nm^{-1} and inset TEM iamges have a width of (a) 100 nm, (b) 200 nm, (c) 700 nm and (d) 400 nm.

S8. SPXRD lattice parameter results and comparison with SAED

Lattice parameters of (Sm,Y)S-TaS_2 obtained from synchrotron PXRD. A (nm^2) of TaS_2 is the area occupied in the *a-b* plane, given by a_{TaS2}*b_{MLC}.

Y content x'	(Sm,Y)	TaS_2		$[(Sm,Y)S]_{1+y}TaS_2$		1 + y
	a (Å)	a (Å)	A (nm^2)	b (Å)	c (Å)	
0	5.569(1)	3.283(1)	0.1870	5.697(1)	22.536(4)	1.179(1)
0.13	5.546(1)	3.285(1)	0.1870	5.693(1)	22.527(4)	1.185(1)
0.23	5.521(1)	3.288(1)	0.1870	5.688(1)	22.504(4)	1.191(1)
0.36	5.504(1)	3.287(1)	0.1868	5.682(1)	22.479(4)	1.194(1)
0.45	5.497(1)	3.286(1)	0.1866	5.679(1)	22.461(4)	1.196(1)
0.73	5.451(1)	3.292(1)	0.1866	5.668(1)	22.409(4)	1.208(1)
1	5.419(1)	3.300(1)	0.1867	5.658(1)	22.364(4)	1.218(1)

c lattice parameter of (O-T-T) (Sm,Y)S-$(TaS_2)_2$ obtained from both 004 reflection (possible overlap with Y_2S_3) and 008 reflection.

Y content x'	(O-T-T) $[(Sm,Y)S]_{1+y}$-$(TaS_2)_2$	
	c (Å) from 004	c (Å) from 008
0	-	-
0.13	34.53(1)	-
0.23	34.498(7)	34.51(6)
0.36	34.49(1)	-
0.45	34.459(5)	34.33(2)
0.73	34.407(4)	34.38(1)
1	34.336(3)	34.31(1)

Comparison of selected lattice parameters showing the good agreement between SAED and SPXRD. The difference in asymmetry might be linked to the measurement of Nanotubes only in the case of SAED and Nanotubes + Flakes in the case of SPXRD. As the relative abundance of flakes increases with x, the difference between SAED and SPXRD also increases.

**x`**	**(Sm,Y)S-TaS2 c (nm)**		**(Sm,Y)S a (nm)**		**(Sm,Y)S asymmetry / %**	
	**SAED**	**SPXRD**	**SAED**	**SPXRD**	**SAED**	**SPXRD**
0	2.21	2.2536(1)	0.549	0.5569(1)	1.47	2.25
0.13	2.22	2.2527(4)	0.549	0.5546(1)	0.21	2.58
0.36	2.20	2.2479(4)	0.544	0.5504(1)	2.07	3.13
0.73	2.22	2.2409(1)	0.539	0.5451(1)	1.28	3.83
1	2.21	2.2364(4)	0.539	0.5419(1)	-	4.22

S9. Incommensurate-commensurate transition

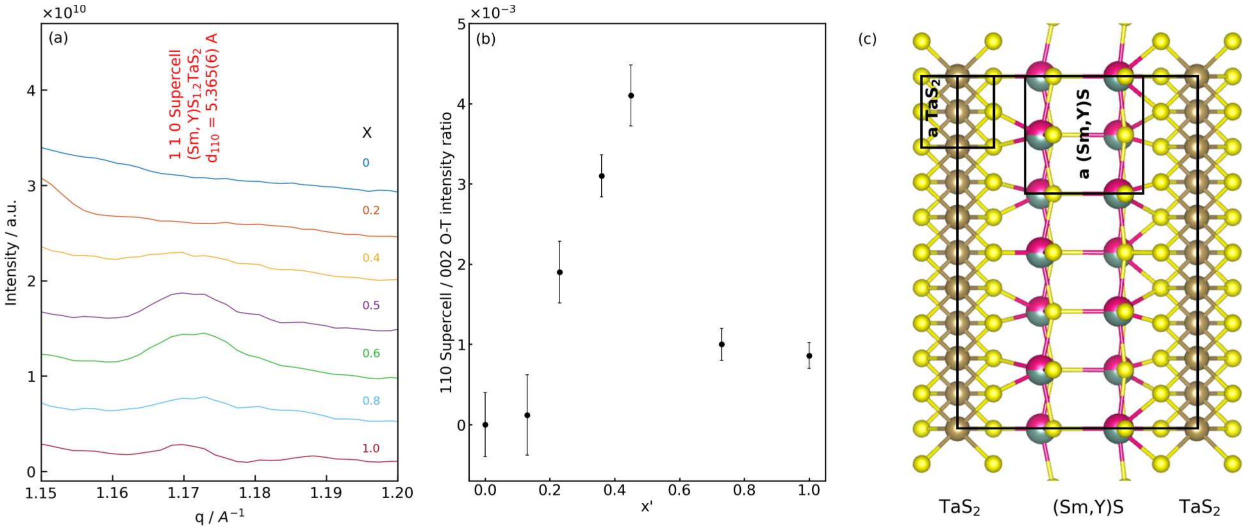

Data and description of incommensurate-commensurate transition. (a). The XRD profile of the supercell $(Sm,Y)_{1.2}TaS_2$ 110 reflection for diffractograms of samples with different Y content x. (b). The integral intensity of the supercell $(Sm,Y)_{1.2}TaS_2$ 110 reflection as a function of Y content x'. (c). Sketch of the proposed supercell $(Sm,Y)_{1.2}TaS_2$ model consisting of 3 (Sm,Y)S and 5 TaS_2 units.

S10. SEM EDS secondary phase

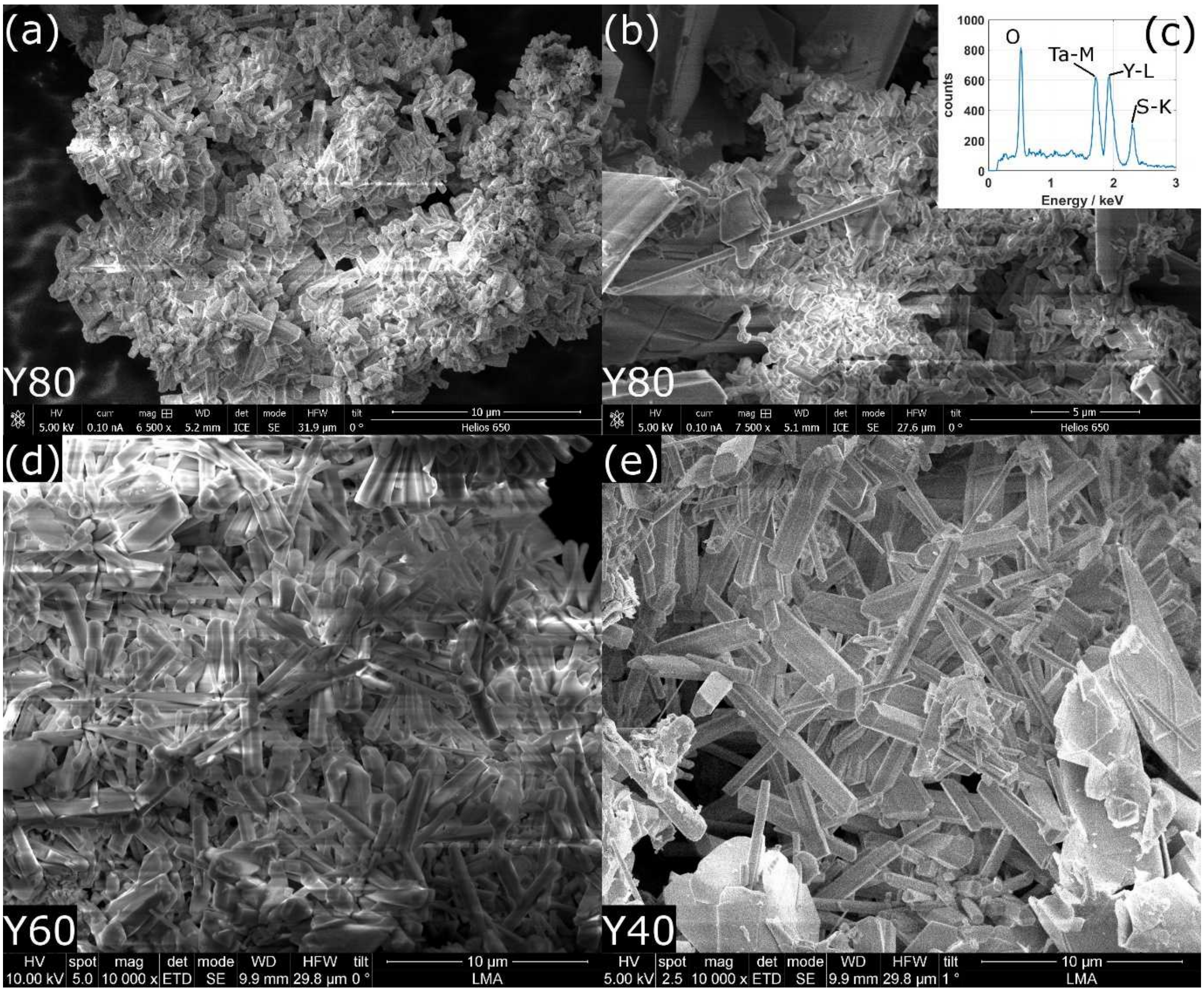

Selected SEM images of the synthesis product showing morphologies clearly identifiable as non-MLC structures for the (a,b) Y80, (d) Y60 and (e) Y40 specimens. SEM-EDS analysis of the secondary phases in the Y80 specimen indicates a composition based on Ta, Y and S (with minimum amount of Sm), which have (partially) oxidized, see exemplary EDS spectrum in (c). The Y60 was prepared from powder dispersion.

S11. Bulk plasmon analysis

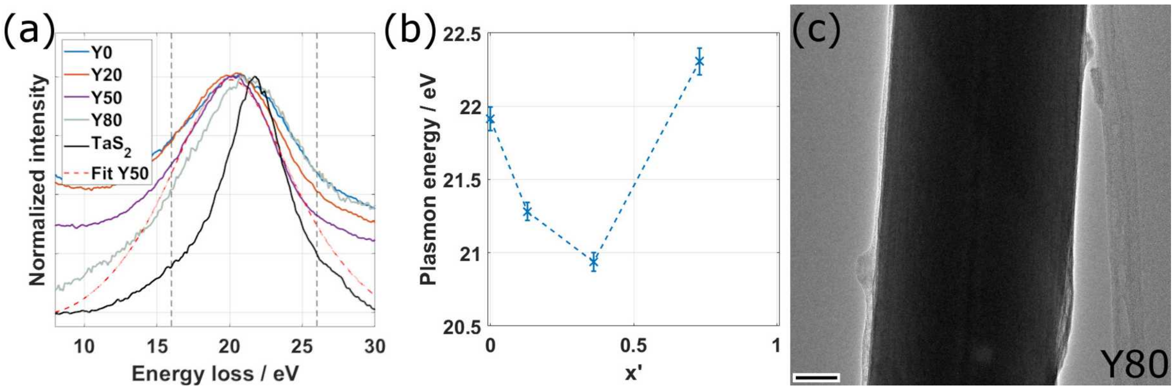

Bulk plasmon analysis conducted by EELS in TEM-diffraction mode for different individual NTs with different composition. (a) EEL spectra of 4 different NTs as indicated compared with the spectrum for TaS_2 (black). The energy loss range used for fitting is indicated by vertical gray lines and the fit to the Y50 spectrum (purple) is shown by a red-dashed line. (b) Determined plasmon energy from the fit to spectra for 3 NTs for each composition plotted over the measured relative Y content x`. Error bar is statistical error on fit only. (c) TEM image of an exemplary NT for the Y80 specimen, scale bar is 40 nm.

S12. Raman spectra

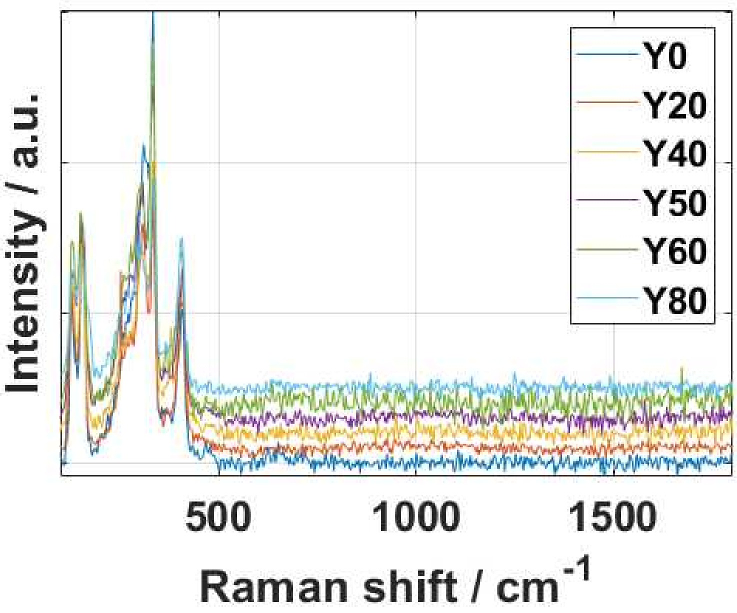

Raman spectra shown in Figure 7a plotted up to high Raman shifts showing the absence of peaks related to oxidized products, typically appearing in the range above 600 cm^{-1}.
